# Tuning PAK Activity to Rescue Abnormal Myelin Permeability in HNPP

**DOI:** 10.1371/journal.pgen.1006290

**Published:** 2016-09-01

**Authors:** Bo Hu, Sezgi Arpag, Xuebao Zhang, Wiebke Möbius, Hauke Werner, Gina Sosinsky, Mark Ellisman, Yang Zhang, Audra Hamilton, Jonathan Chernoff, Jun Li

**Affiliations:** 1 Department of Neurology, Center for Human Genetics Research, Vanderbilt Brain Institute, Vanderbilt University School of Medicine, Nashville, Tennessee, United States of America; 2 Department of Anatomy and Cell Biology, Wayne State University, Detroit, Michigan, United States of America; 3 Center Nanoscale Microscopy and Molecular Physiology of the Brain (CNMPB), GÖttingen, Germany; 4 National Center for Microscopy and Imaging Research, University of California, San Diego, La Jolla, California, United States of America; 5 Cancer Biology, Fox Chase Cancer Center, Philadelphia, United States of America; 6 Tennessee Valley Healthcare System, Nashville, Tennessee, United States of America; The Jackson Laboratory, UNITED STATES

## Abstract

Schwann cells in the peripheral nervous systems extend their membranes to wrap axons concentrically and form the insulating sheath, called myelin. The spaces between layers of myelin are sealed by myelin junctions. This tight insulation enables rapid conduction of electric impulses (action potentials) through axons. Demyelination (stripping off the insulating sheath) has been widely regarded as one of the most important mechanisms altering the action potential propagation in many neurological diseases. However, the effective nerve conduction is also thought to require a proper myelin seal through myelin junctions such as tight junctions and adherens junctions. In the present study, we have demonstrated the disruption of myelin junctions in a mouse model (*Pmp22*+/-) of hereditary neuropathy with liability to pressure palsies (HNPP) with heterozygous deletion of *Pmp22* gene. We observed a robust increase of F-actin in *Pmp22*+/- nerve regions where myelin junctions were disrupted, leading to increased myelin permeability. These abnormalities were present long before segmental demyelination at the late phase of *Pmp22*+/- mice. Moreover, the increase of F-actin levels correlated with an enhanced activity of p21-activated kinase (PAK1), a molecule known to regulate actin polymerization. Pharmacological inhibition of PAK normalized levels of F-actin, and completely prevented the progression of the myelin junction disruption and nerve conduction failure in *Pmp22*+/- mice. Our findings explain how abnormal myelin permeability is caused in HNPP, leading to impaired action potential propagation in the absence of demyelination. We call it “functional demyelination”, a novel mechanism upstream to the actual stripping of myelin that is relevant to many demyelinating diseases. This observation also provides a potential therapeutic approach for HNPP.

## Author Summary

Demyelination has been widely regarded as one of the most important mechanisms impairing the nerve conduction of electrical impulses in many neurological diseases. Failure of the nerve conduction results in sensory and motor deficits. In the present study, we investigated a novel mechanism that also impairs nerve conduction without the removal of myelin, called functional demyelination. Deletion of one of two copies of the *PMP22* gene causes hereditary neuropathy with liability to pressure palsies (HNPP). Our recent study reports that deficiency of PMP22 in HNPP disrupts myelin junctions of Schwann cells that seal the spaces between layers of myelin membrane. This disruption leads to “leaky” myelin that impairs the conduction of electrical impulses on the nerves. In the present study, using a HNPP mouse model (*Pmp22*+/-), we have identified a molecular pathway responsible for the disruption of myelin junctions. Our results showed an increase of actin polymerization in the areas where myelin junctions resided. This increase was associated with an up-regulation of PAK1 activity, a kinase function that is known to regulate actin polymerization. We thus treated the HNPP mice with PAK1 inhibitor. This treatment completely prevented the progression of nerve conduction failure and HNPP pathology. This work offers a promising therapeutic approach for HNPP. Moreover, myelin junction disruption takes place long before the actual stripping of myelin (demyelination) in the late phase of HNPP. Therefore, our finding provides a mechanism upstream to segmental demyelination, a pathological process relevant to many demyelinating diseases.

## Introduction

Depolarizing current at the node of Ranvier is typically five times higher than the minimum required to trigger the action potential. This surplus is called the “safety factor” [1]. The safety factor is secured by the wrapping of the glial cell membrane around axons to produce the insulating sheath, called myelin. Schwann cells in the peripheral nervous system form the myelin during early development. However, the maturation process may extend over the first 5 years of human life when the conduction speed of action potential reaches the level of adulthood. The safety factor resulted from the insulation of myelin may be impaired in a variety of neurological diseases, usually as a result of the removal of myelin (demyelination). Denuded axons shunt the depolarizing current out of nerve fibers, leading to either a reduction of conduction velocity or complete failure of action potential propagation. The latter is called “conduction block” and produces focal sensory loss and/or limb paralysis [2, 3].

**Fig 1:**
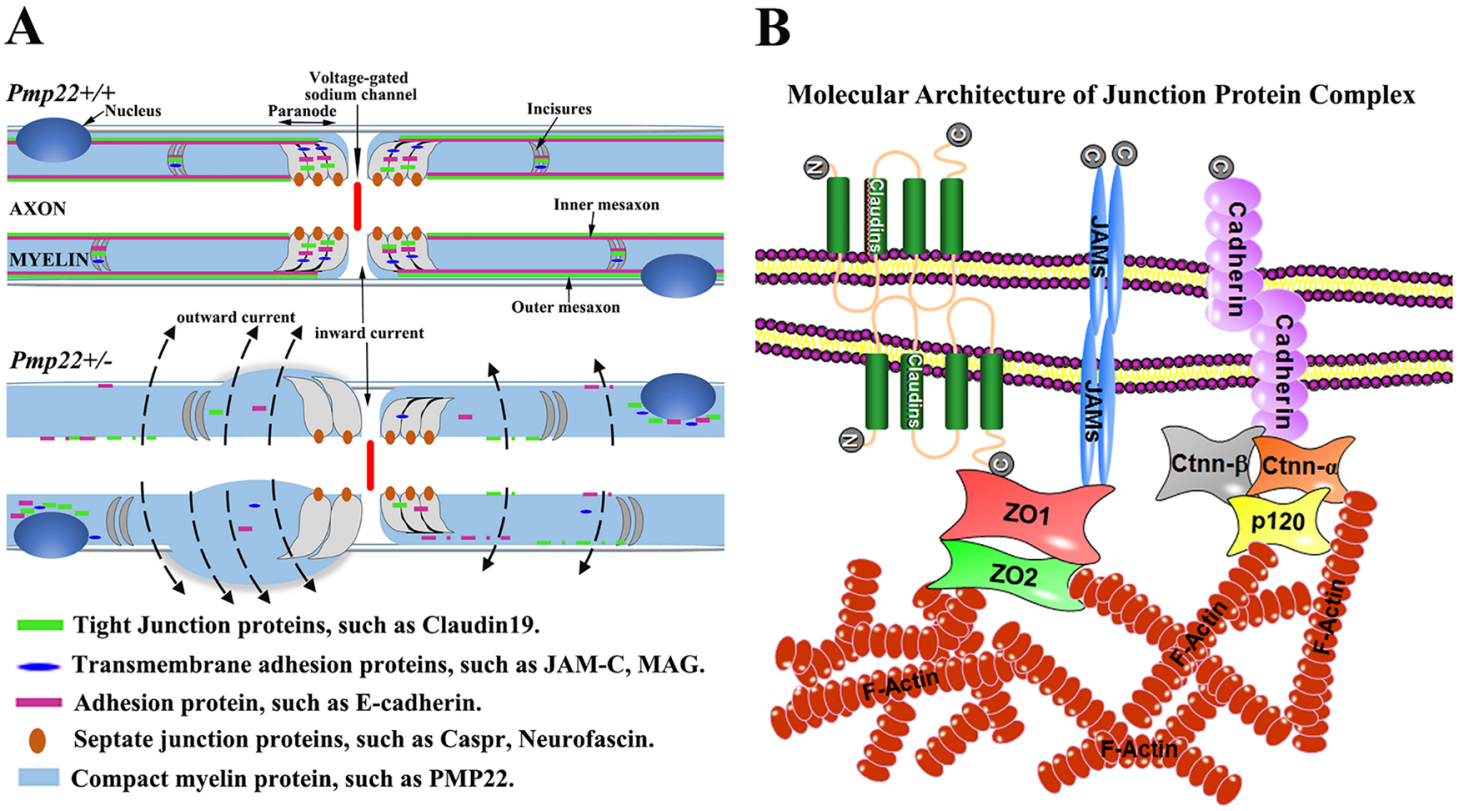
Schematic illustration of myelin junction disruption. **(A)** Mechanism of functional demyelination (modified from Guo et al, Ann Neurol 2014): Myelin junctions in *Pmp22*+/+ nerve are in non-compact myelin regions, including paranodes, incisures and mesaxons. These junctions seal the spaces between myelin lamina. A *Pmp22*+/- nerve fiber is depicted and develops a tomaculae in the left paranode extending into juxtaparanode and internode, but there is no segmental demyelination. However, junction protein complexes are disrupted or disappeared in the non-compact myelin. These junction proteins may be found in aberrant locations, including perinuclear areas or tomaculous myelin. Abnormal junctions in *Pmp22*+/- nerves increase myelin permeability (or increase of capacitance). **(B)** Molecular architecture of junction protein complex: Transmembrane proteins establish "trans-adhesion" between opposing membranes. Through adaptor proteins, such as ZO1/2 or catenins, these junction protein complexes are stabilized by sub-membrane actin networks. Alteration of the actin network has been shown to disassemble junctions in epithelial cell models [29, 30].

Although demyelination is widely regarded as one of the most important mechanisms altering the safety factor, effective nerve conduction is also thought to require a proper myelin seal through myelin junctions such as tight junctions and adherens junctions. These junctions seal the spaces between adjacent myelin lamellae and between the myelin and axolemma [4]. We have observed excessively permeable myelin (i.e., an increase of capacitance) in a mouse model of hereditary neuropathy with liability to pressure palsies (HNPP) due to disruption of these myelin junctions. This novel mechanism impairs action potential propagation in the absence of demyelination [5]. We call it “functional demyelination” (Fig 1A). Thus, this mechanism denotes pathological processes that may alter the insulating quality of myelin without physically stripping off the myelin sheath.

HNPP is caused by a heterozygous deletion of *PMP22* gene in human chromosome 17p12. *PMP22* encodes a tetra-span membrane protein primarily expressed in peripheral nerve myelin [6–8]. Mice with heterozygous knockout of *Pmp22* recapitulate the pathology of humans with HNPP, including tomacula with excessive myelin decompaction that extends from paranodes to juxtaparanodes and internodes [9]. Application of mechanical compression on *Pmp22*+/- mouse nerves induced conduction block (i.e., failure of action potential propagation) more rapidly than that in *Pmp22*+/+ nerves. This finding is consistent with the key clinical features in patients with HNPP—focal sensory loss and weakness when nerves are exposed to mild mechanical stress [10, 11]. Therefore, these mice have become an authentic model of HNPP.

There are three types of junctions in myelin: tight junctions, adherens junctions, and septate junctions [4]. All are mainly in non-compact myelin regions (Fig 1A): paranodal loops, Schmidt-Lanterman incisures (SLI), and inner/outer mesaxons [12]. Although each type of junctions has distinct protein constituents, they share similar molecular architectures (Fig 1B). For instance, tight junctions are formed by polymerization of claudins, a family of tetraspan membrane proteins. C-terminals of claudins interact with a group of cytoplasmic adaptors such as ZO1 or ZO2 [13]. These PDZ-containing proteins directly interact with actins and link the tight junctions to the cytoskeleton for stabilization [14]. Adherens junctions employ a similar organization. E-cadherin has a glycosylated extracellular domain, a single transmembrane domain, and a cytoplasmic c-terminal tail that interacts with adaptor catenins (α-catenin, β-catenin and p120/σ-catenin). α-catenin directly interacts with actin filaments. The actin network is subject to the regulation of small GTPases (Cdc42 or Rac1) and their effectors such as p21-activated kinase (PAK1) [14].

Furthermore, all junctions are strengthened by a group of Ig-domain proteins, such as JAM-C in myelin, that form transmembrane dimers juxtaposed to the junctions to seal the space between the opposing membranes [15]. Because actin networks are involved in the stabilization of all junctions, we test a hypothesis that PMP22 deficiency disrupts myelin junctions by altering actin polymerization.

## Results

### Disruption of myelin junctions takes place long before segmental demyelination seen in the late stage of *Pmp22*+/- mice

**Fig 2:**
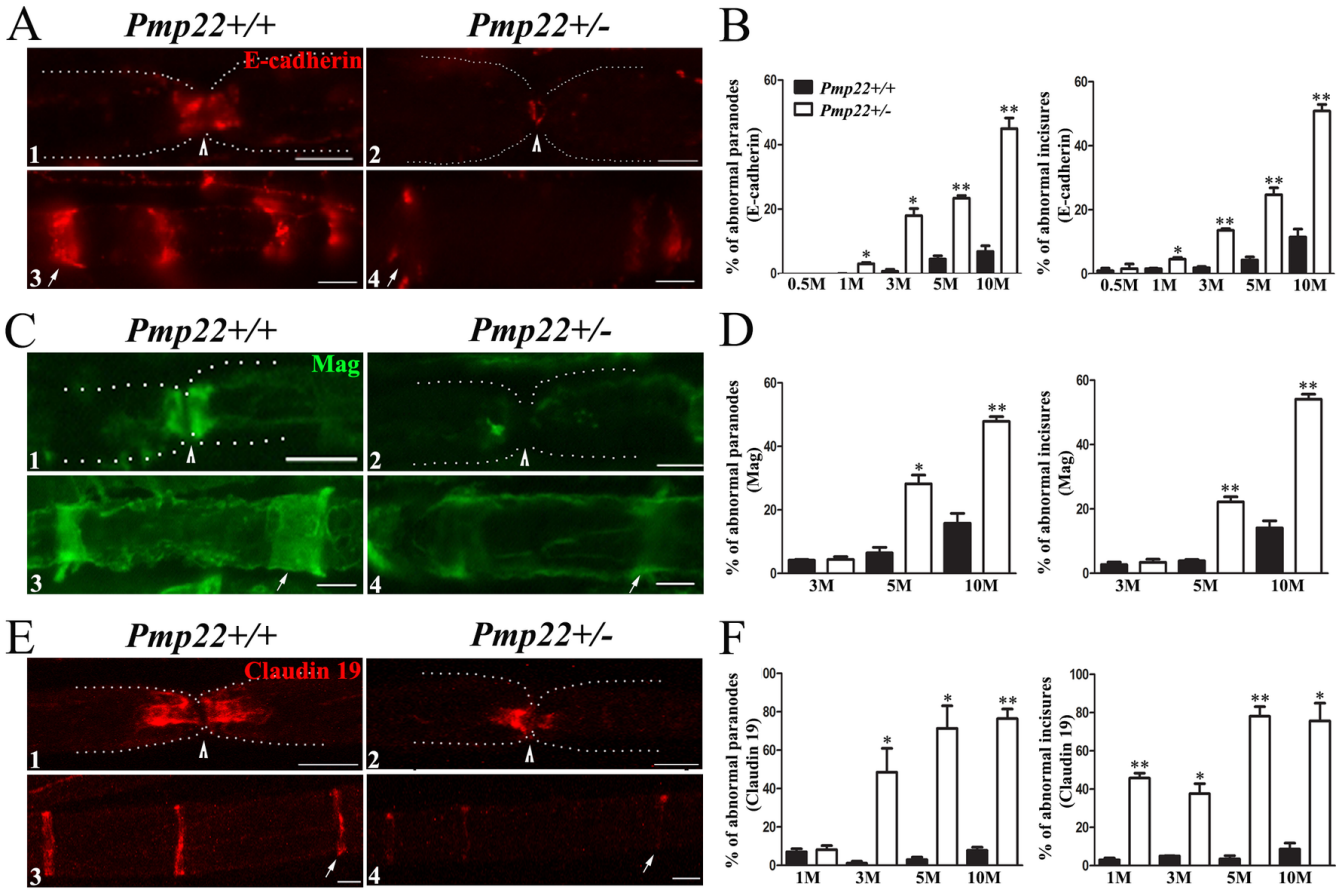
Disruption of myelin junctions in *Pmp22+/-* nerves during aging. Paraffin sections of mouse sciatic nerves were stained with antibodies. Percentages of abnormal paranodes or incisures were manually counted [5]. An abnormal paranode or incisurae was defined as the staining was absent in more than a half of normally stained paranodal or incisure territory. **(A)** E-cadherin antibody stained paranodes (red-color stained areas adjacent to the node marked by an arrowhead in **A1**) in 3-month-old *Pmp22*+/+ mouse nerve fiber but showed no signal in *Pmp22*+/- paranodes (**A2**). White dots outline the margin of the nerve fiber, based on its phase-contrast image. A strong E-cadherin band at the *Pmp22*+/- node (arrowhead in **A2**) was presumably due to an ectopic expression in Schwann cell microvilli. E-cadherin antibodies also stained *Pmp22*+/+ incisures (arrow in **A3**) but showed minimal signals in *Pmp22*+/- incisures (arrow in **A4**). Scale bars = 10μm. **(B)** There was a significant increase of abnormal E-cadherin-stained paranodes and incisures from 1 month of age onward (n = 140–340 paranodes and 800–1,700 incisures from either 3 *Pmp22+/+* or 3 *Pmp22+/-* mice at each age group). *P < 0.01, ** *P* < 0.0001. **(C)** Mag staining was present in the paranodes (**C1**) and incisures (arrow in **C3**) of 5-month-old *Pmp22+/+* nerves but decreased in *Pmp22*+/- paranodes (**C2**) or incisures (arrow in **C4**). Scale bars = 10μm. **(D)** There was a significant increase of abnormal Mag-stained paranodes or incisures from 5 month of age onward (n = 160–300 paranodes and 900–1,500 incisures from 3 *Pmp22+/+* and 3 *Pmp22+/-* mice at each age group). *P < 0.01, ** *P* < 0.0001. **(E)** Teased sciatic nerve fibers of *Pmp22*+/+ mice at the 3 months of age were stained with antibodies against claudin-19 to show paranodes (**E1)** and incisures (arrow in **E3**). The staining was reduced in *Pmp22*+/- paranodes (**E2**) and incisures **(E4**). Scale bars = 10μm. **(F)** There was a significant difference found in paranodes from 3 months of age onward and in incisures from 1 month of age onward (n = 280–380 paranodes and 800–1,200 incisures from 3 *Pmp22+/+* and 3 *Pmp22+/-* mice at each age group). *P < 0.01, ** *P* < 0.0001.

While we have described the disruption of myelin junctions in *Pmp22*+/- nerves [5], the disruption had yet been evaluated during aging. In this study, we found dislocation of E-cadherin (marker for adherens junction), Mag (marker for transmembrane protein of paranodal loop), and claudin-19 (marker for tight junction) in *Pmp22*+/- paranodes and incisures from 2 weeks to 10 months of age (Fig 2). Under electron microscopy, junction abnormalities with paranodal lamina splitting were qualitatively observed in *Pmp22*+/- nerves (S1 Fig). In line with our previous studies [5, 10], localization of Caspr and neurofascin at septate junctions was unchanged (S2 Fig). Note that the total amounts of these junction proteins were not altered in *Pmp22*+/- nerves by Western blot [5]. Taken together, myelin junctions in *Pmp22*+/- nerves were abnormally formed during development and disrupted in adulthood. This abnormality was observable as early as 2 weeks, which was months ahead of segmental demyelination seen only after 10–12 months of age [5, 10].

Results above predict action potential propagation failure in a subset of *Pmp22*+/- nerve fibers with no segmental demyelination but severely increased myelin permeability [1]. We performed nerve conduction studies (NCS) in mice at ages of 2, 6, and 12 months. There was a significant reduction of compound muscle action potential (CMAP) amplitudes in all age-groups of *Pmp22*+/- mice compared with those in *Pmp22*+/+ mice (S1 Table). In contrast, conduction velocities were not altered in *Pmp22*+/- nerves. To determine whether the decrease of CMAP was due to axonal loss, we performed sciatic nerve morphometric analysis at ages of 1, 3 and 6 months. The numbers of myelinated nerve fibers were not significantly different between *Pmp22*+/+ and *Pmp22*+/- mice (S2 Table). These findings do not support axonal loss.

**Fig 3:**
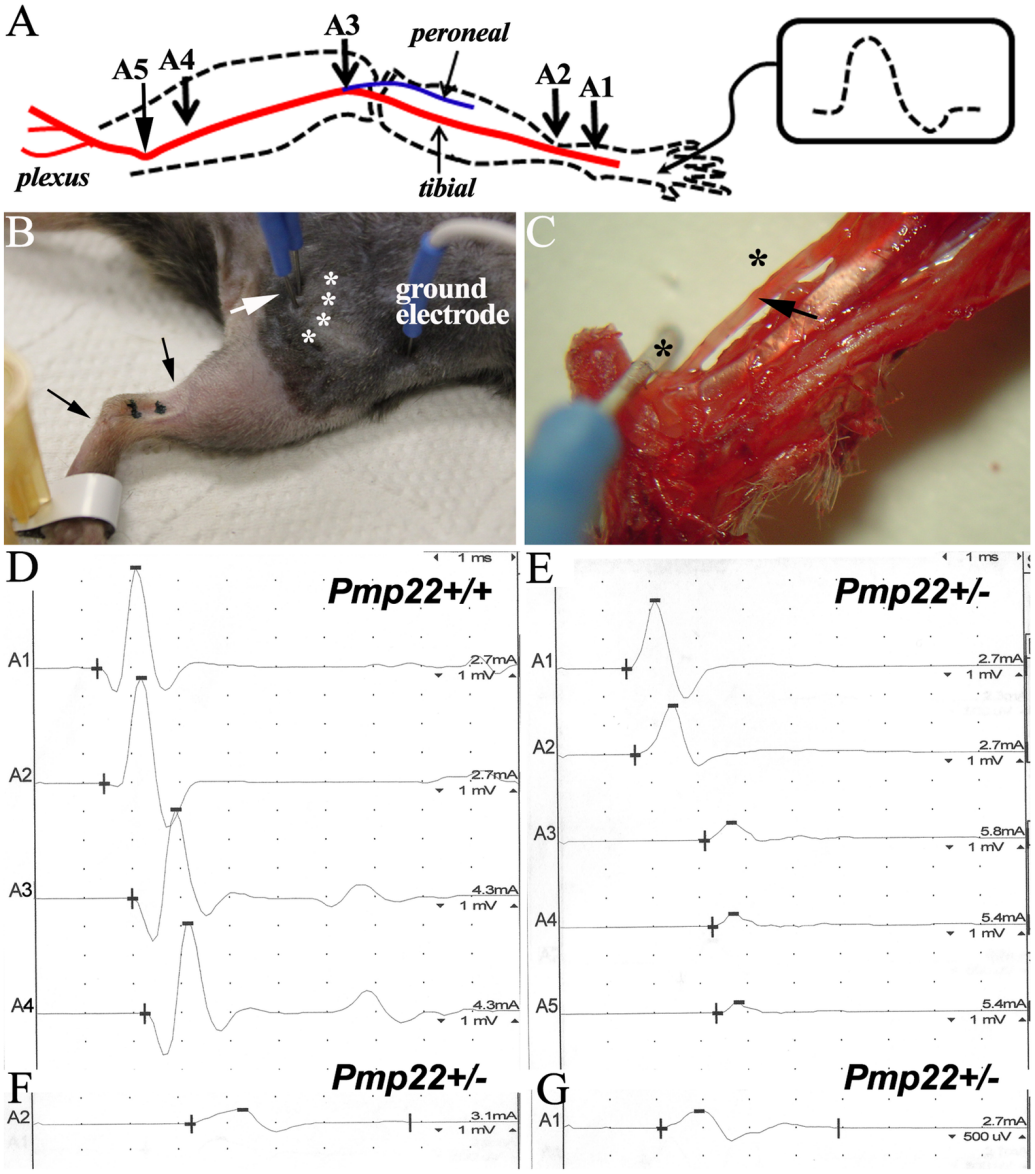
Conduction block was detected in naïve *Pmp22*+/- nerves. **(A)** A diagram shows the setting for the experiments. A1-5 indicates the sites where the stimulation electrodes were placed on surgically exposed sciatic nerve. **(B)** In conventional NCS, proximal stimulation electrode is inserted blindly into the sciatic notch (white arrow in B). Variations of distances between the electrode and sciatic nerve (array of white asterisks) are not avoidable. This variation was eliminated by surgically exposing the sciatic nerves. Two black dots indicate the sites where distal stimulation electrodes were placed around ankle. **(C)** Area nearby ankle was dissected to reveal the tibial nerve (arrow in C). Due to the tiny space of this area, distance between the electrode and tibial nerve was highly consistent (two asterisks represent the sites of black dots in B). Thus, it did not require surgical exposure to place the distal stimulation electrodes. Note that needle electrode at the asterisk sites was inserted just through the dermis to avoid any nerve injury. **(D)** CMAP amplitudes were similar between A1 to A4. **(E)** CMAP in a *Pmp22*+/- mouse at A3-A5 showed a >50% reduction of the A2 amplitude. This finding demonstrated a conduction block that was defined as a ≥50% decrease of proximal CMAP amplitude over the distal CMAP amplitude, a stringent criterion used in human NCS [41]. Conduction block was found in 12 out of 17 studied *Pmp22*+/- mice, but not in *Pmp22*+/+ mice. (**F**) CMAP was recorded from a different mouse and showed a distal latency (3.3ms) 2 times longer than that (1.2ms) in *Pmp22*+/+ nerve (A2 in D). The doubled distal latency was found in 2 mice out of the 17 *Pmp22*+/- mice, while the remaining 15 mice had variable degrees of prolonged distal latency. **(G)** CMAP in this mouse had a duration of 4ms (temporal dispersion) that was about twice longer than that in *Pmp22*+/+ nerve (A1 in D). In average, the CMAP duration in 17 *Pmp22*+/- mice (3.9±1.7ms) was significantly longer than that in 7 *Pmp22*+/+ mice (2.3±0.4ms; p = 0.001; 3–10 month old).

To directly evaluate conduction block, sciatic nerves were surgically exposed to eliminate technical variations in NCS (Fig 3A, 3B and 3C). Conduction block (Fig 3D and 3E) was detected in 12 out of 17 studied *Pmp22*+/- mice but not found in any *Pmp22*+/+ mice. The remaining 5 *Pmp22*+/- mice had at least one of the three abnormalities—prolongation of distal latency (Fig 3F), temporal dispersion (Fig 3G), or both. Therefore, conduction block was present in *Pmp22*+/- nerves. This explains the decrease of CMAP amplitudes in *Pmp22*+/- mice.

### F-actin is increased in *Pmp22*+/- nerves and co-localized with myelin junctions

**Fig 4:**
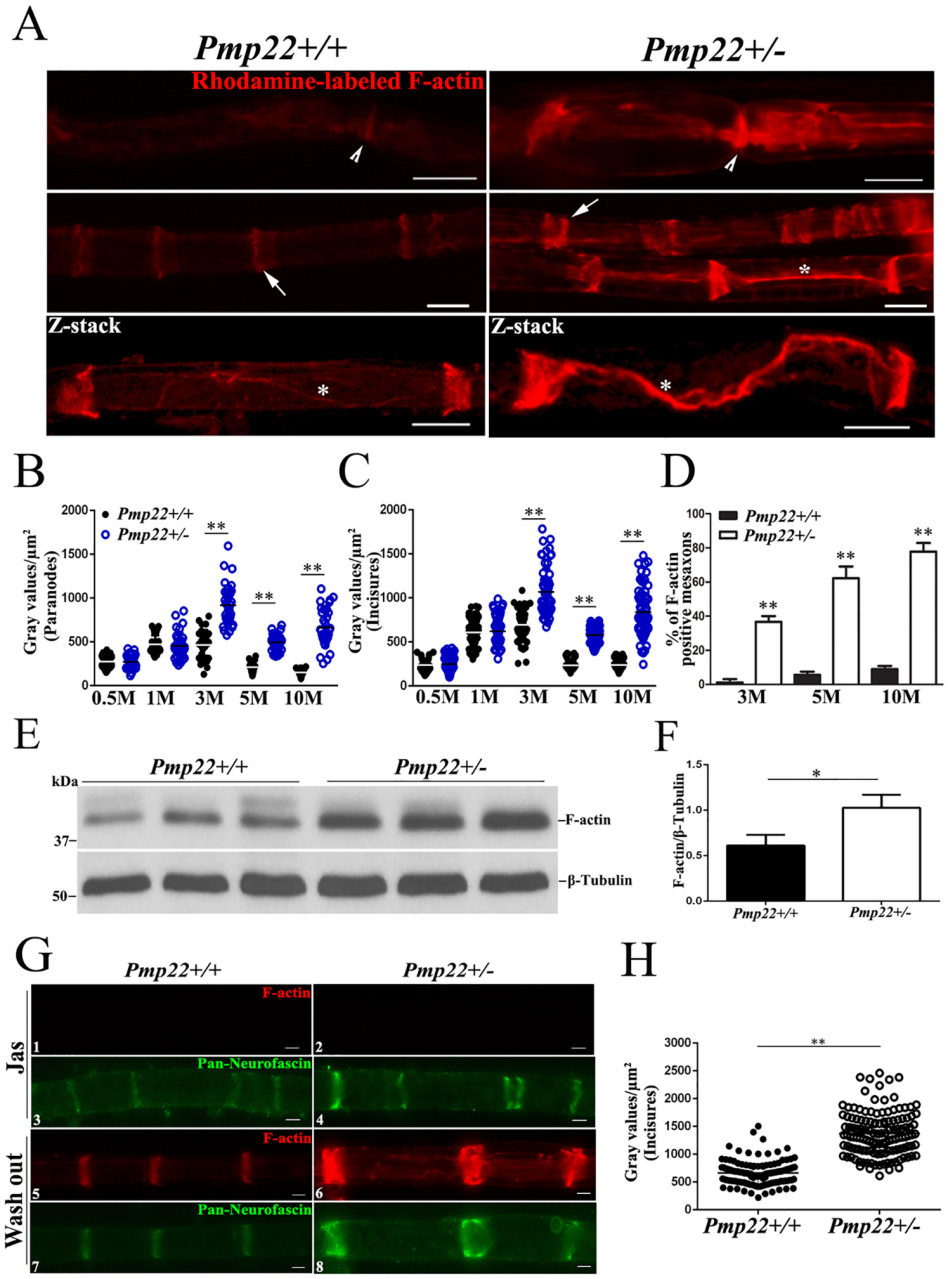
Abnormally increased actin polymerization in the regions where myelin junctions reside. **(A)** Teased nerve fibers of mouse sciatic nerves were stained with fluorescent phalloidin, which was localized at nodes (arrowheads), incisures (arrows) and mesaxons (asterisks). F-actin was strongly expressed in *Pmp22+/-* nerves. Images in the 3^rd^ row were taken under confocal microscopy. The maximal projection of z-stack images was presented to show the mesaxon changes of F-actin at different layers. Scale bars = 10μm. **(B-C)** Fluorescence intensity was quantified by placing 2.5μm x 2.5μm interest box 10μm away from the node of Ranvier and by including the entire area of every incisures. The intensity of F-actin staining was increased in *Pmp22*+/- paranodes and incisures from 3 months of age onward (n = 40–50 paranodes, 60–70 incisures from 3 *Pmp22+/+* and 3 *Pmp22+/-* mice at each age group). ** *P* < 0.0001; M = month. **(D**). The mesaxons with clearly visible F-actin-staining (asterisk in **A**) were counted in teased nerve fibers of *Pmp22+/+* and *Pmp22+/-* mice. The F-actin stained mesaxons in *Pmp22*+/- mice were increased from 3 month of age onward (n = 75 mesaxons from 3 *Pmp22+/+* and 3 *Pmp22+/-* mice at each age group). ** *P* < 0.0001; M = month. **(E)** Western blot analysis of F-actin was performed in the sciatic nerves of 3 month-old *Pmp22+/+* and *Pmp22+/-* mice. **(F)** The levels of F-actin were significantly increased in *Pmp22+/-* nerves, compared with those in *Pmp22+/+* nerves. *P < 0.05. **(G)** *Pmp22+/+* and *Pmp22+/-* sciatic nerve explants were cultured for 3 hours in the presence of jasplakinolide (Jas) and double-stained with fluorescent phalloidin and an anti-Pan-Neurofascin antibody to label incisures. A group of explants was washed following Jas treatment and cultured for another 6 hours in jasplakinolide-free medium. The newly formed F-actin was strongly increased in **G6**. Scale bars = 10μm. **(H)** Fluorescence intensity was quantified by including the entire area of each incisures. The intensity of new F-actin was increased in 3 month-old *Pmp22*+/- incisures, compared with those in *Pmp22*+/+ nerve fibers (n = 120 incisures from 3 *Pmp22+/+* and 3 *Pmp22+/-* mice; Scale bars = 5μm). ** *P* < 0.0001.

F-actin is a common “denominator” in all types of junction complexes (Fig 1B) for junction stabilization [14]. We speculated an altered actin polymerization in PMP22 deficiency. Teased mouse nerve fibers were stained with rhodamine-conjugated phalloidin known to specifically label F-actin [16]. F-actin was localized in non-compact myelin regions (Fig 4A) where myelin junctions also reside (Figs 1 and 2). Quantification of F-actin fluorescence intensity showed a significant difference between *Pmp22*+/+ and *Pmp22*+/- nerves from 3 to 10 months of age (Fig 4B, 4C and 4D). Moreover, Western blot confirmed the increase of F-actin in *Pmp22*+/- nerves (Fig 4E and 4F).

We tested dynamics of F-actin formation as described [16]. Jasplakinolide is a membrane permeable cyclo-depsipeptide that competes with phalloidin for F-actin binding. After saturating the existing F-actin with jasplakinolide, phalloidin only labeled newly formed F-actin. *Pmp22*+/- nerves showed a higher level of new F-actins than that in *Pmp22*+/+ nerves (Fig 4G and 4H).

### Activity of PAK1 is increased in *Pmp22*+/- nerves

**Fig 5:**
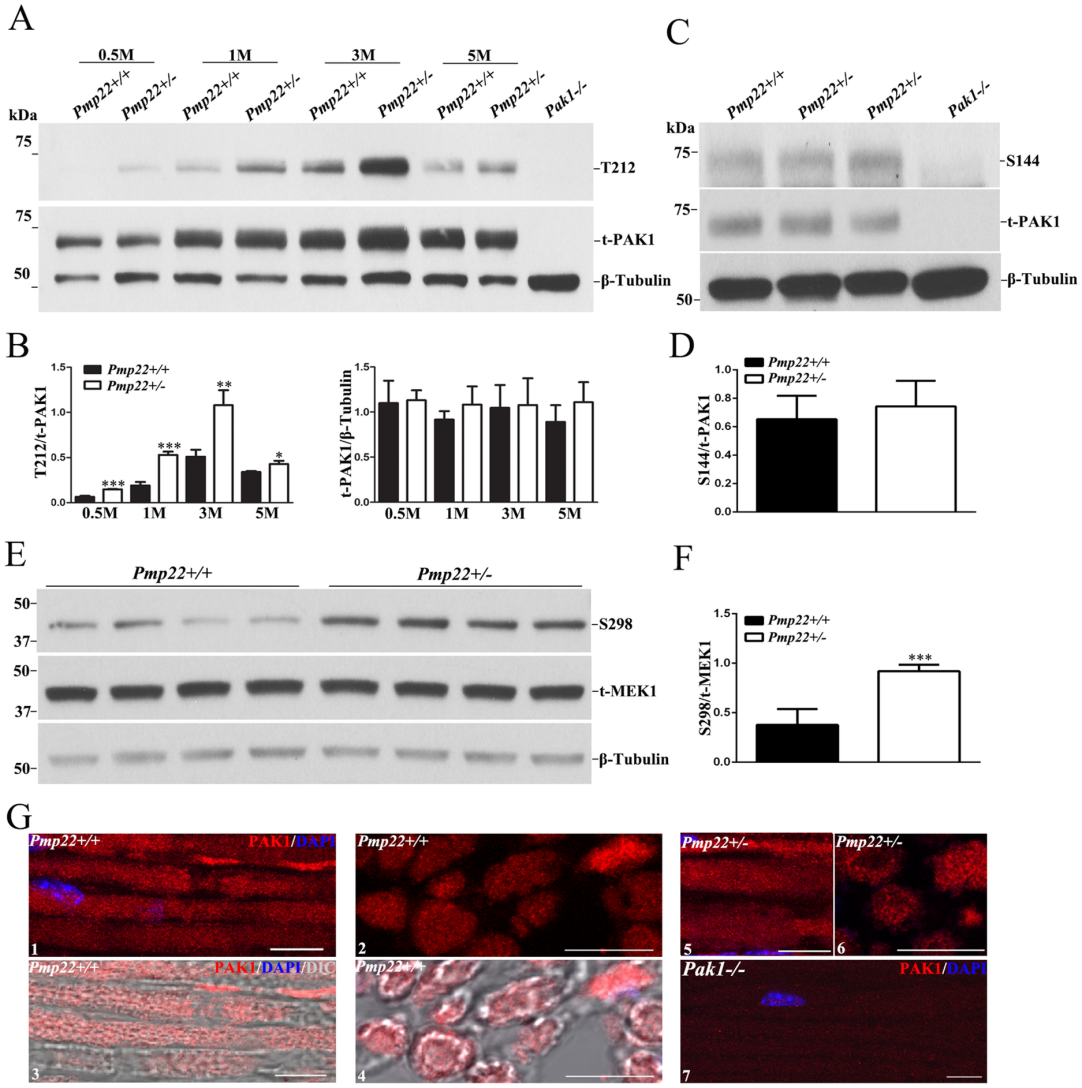
PAK1 activity is increased in *Pmp2*2+/- nerves. **(A)** Western blot of phosphorylated PAK1 (T212) and total PAK1 (t-PAK1) in the sciatic nerves from 0.5–5 month-old *Pmp22+/+* and *Pmp22+/-* mice. Both T212 and t-PAK1 were not detectable in the sciatic nerves of *Pak1*-/- mice (line 9). **(B)** T212 level was normalized against t-PAK1 levels. T-PAK1 level was normalized against β-Tubulin levels. The levels of T212, but not t-PAK1 levels, were significantly increased in *Pmp22+/-* nerves, compared with those in *Pmp22+/+* nerves. *P < 0.05, ** *P* < 0.01, *** *P* < 0.001. **(C)** Western blot of S144 in the sciatic nerves of 3 month-old *Pmp22+/+* and *Pmp22+/-* mice. S144 were not detectable in the sciatic nerves of *Pak1*-/- mice (line 4). **(D)** S144 levels were normalized against t-PAK1 levels. S144 level was not significantly different between *Pmp22+/+* and *Pmp22+/-* nerves. **(E)** Western blot for phosphorylated MEK1 (S298) and total MEK1 (t-MEK1) in the sciatic nerves of 3 month-old *Pmp22+/+* and *Pmp22+/-* mice. **(F)** S298 levels were normalized against t-MEK1 levels. S298 levels were significantly increased in *Pmp22+/-* nerves, compared with those in *Pmp22+/+* nerves. *** *P* < 0.001. **(G)** Longitudinal (G1, G5) and transverse (G2, G6) sections of sciatic nerves were stained with antibodies against PAK1. The staining was superimposed with phase-contrast images (G3, G4), which showed PAK1 located in myelin and axons. PAK1 were not detectable in the sciatic nerves of *Pak1*-/- mice (G7). Scale bars = 10μm.

Alteration of actin polymerization prompted us to examine changes of F-actin’s regulators such as Cdc42, Rac1, and PAK1 [17]. Both Cdc42 and Rac1 are functionally essential. Removal of either Cdc42 or Rac1 results in severe dysmyelination [18], which makes the two molecules unfavorable targets of intervention. In contrast, constitutive knockout of PAK1 (*Pak1*-/-) produces negligible phenotype in mice [19]. We have also confirmed normal morphology, electrophysiology and functions in *Pak1*-/- peripheral nerves (S3 Fig). Yet, this kinase has been shown to play roles in actin polymerization and cellular focal adhesion [17]. In mouse sciatic nerves, we detected PAK1 (Fig 5A) and PAK2 but not PAK3 (S4 Fig). Levels of total PAK1 (Fig 5A and 5B) or PAK2 (S4 Fig) were not different between *Pmp22*+/+ and *Pmp22*+/- nerves. Immunostaining with antibodies against total PAK1 showed diffuse distribution in the sciatic nerves similarly between *Pmp22*+/+ and *Pmp22*+/- mice (Fig 5G).

PAK1-3 activation involves autophosphorylation at multiple amino acid residues, including S144, S199, and/or T423 [17]. In addition, PAK1, not PAK2-3, can be phosphorylated at T212 to activate PAK1 independently of small GTPases and regulate F-actin formation [20]. Western blot of sciatic nerve lysates showed a significantly increased level of T212 (but not S144) in *Pmp22*+/- nerves compared with that in *Pmp22*+/+ nerves (Fig 5A, 5B, 5C and 5D). The activity of pPAK1 reached its peak at age of 3-month-old, which correlated with the time when tomacula are actively formed [10]. We verified specificity of total PAK1, S144, and T212 antibodies using *Pak1*+/+ and *Pak1*-/- nerves. All antibodies detected PAK1 in *Pak1*+/+ nerves but not in *Pak1*-/- nerves (Fig 5A and 5C).

PAK1 has been shown to phosphorylate MAPK kinase-1 (MEK1) at its S298 residue [21]. Western blot revealed an increase of phosphorylated MEK1 in *Pmp22*+/- nerves compared with that in *Pmp22*+/+ nerves (Fig 5E and 5F). Together, these data support an increase of PAK1 activation in PMP22 deficient nerves. Residues in other PAKs could be phosphorylated but cannot be substantiated due to the lack of specific antibodies.

### PAK1 is associated with junction protein complexes

**Fig 6:**
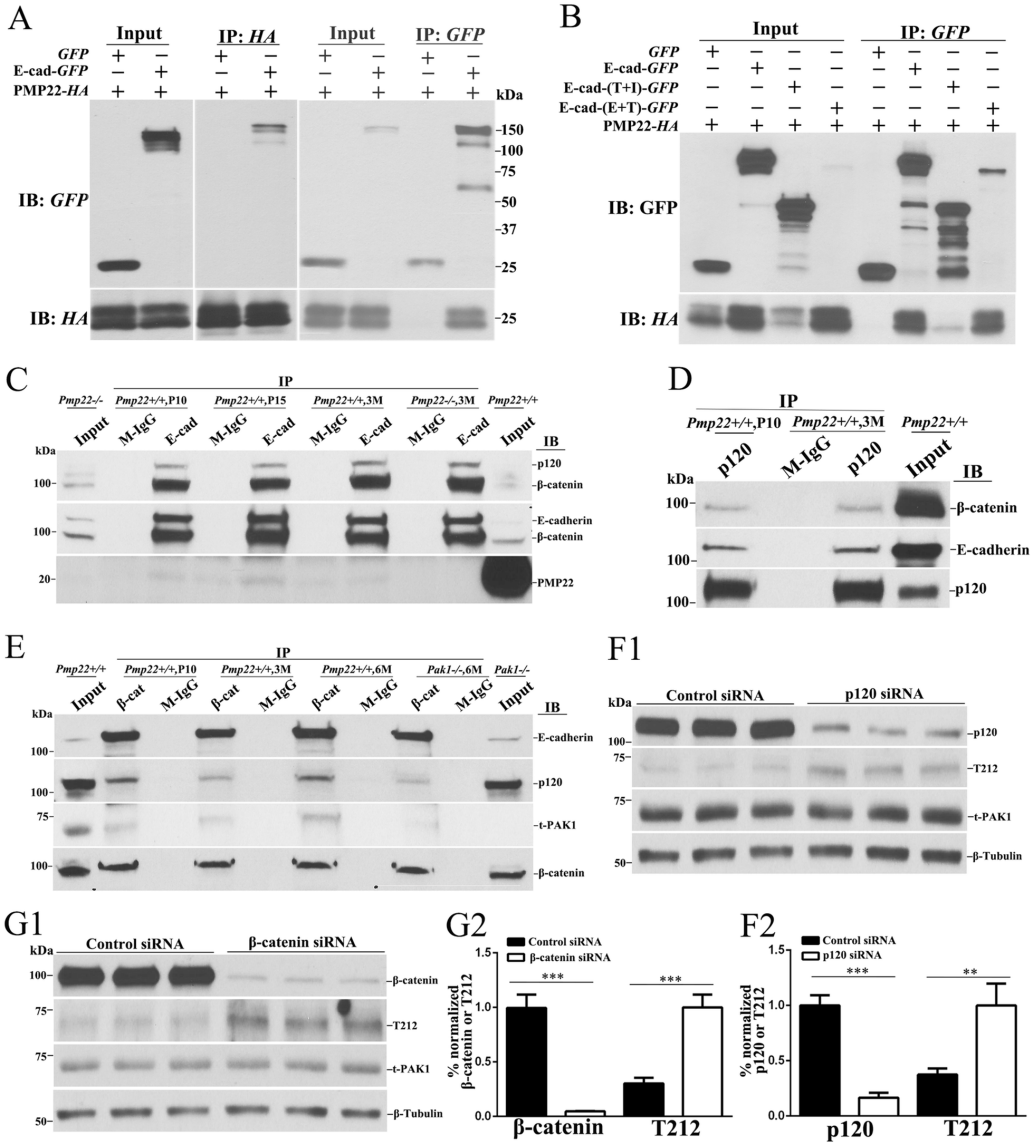
PAK1 complexes with adherens junction proteins and is activated after junction complex is disrupted. **(A)** Human HA-tagged PMP22 was co-expressed with GFP or GFP-tagged E-cadherin in HEK293a cells. Cell lysates were loaded as inputs and blotted with the anti-HA or anti-GFP antibodies (Input lanes). Lysates were immunoprecipitated and blotted with GFP or HA antibodies (IP lanes). **(B)** GFP or GFP-tagged wild-type E-cadherin and mutants were co-expressed with HA-tagged PMP22 in HEK293a cells. Lysates were subjected to co-IP. IB = immunobltting; IP = immunoprecipitation. E+T = mutant with intracellular domain deleted, T+I = mutant with extracellular domain deleted. **(C)** Lysates were extracted from mouse sciatic nerves at postnatal day 10, day 15 and 3 month-old *Pmp22+/+* mice. Lysates were immunoprecipitated with anti-E-cadherin antibody and the precipitated endogenous proteins were blot with anti-PMP22, anti-β-catenin and anti-p120/σ-catenin antibody. E-cadherin antibodies were able to pull down PMP22 in P10 and 15 days *Pmp22+/+* nerves, but failed to do so in 3-month-old *Pmp22+/+* and *Pmp22-/-* nerves (negative control). Also, E-cadherin antibodies were able to pull down β-catenin and σ-catenin in *Pmp22+/+* nerves. IgG was used as another negative control. Note that the band of PMP22 was around 22kDa, suggesting a major portion of the PMP22 proteins were glycosylated. **(D)** IP using control IgG and anti-p120/σ-catenin antibody was carried out in extracts from mouse sciatic nerves at P10 and 3 month-old *Pmp22+/+* mice. The presence of β-catenin and E-cadherin in these IP was evaluated by immunoblotting. The p120/σ-catenin antibodies were able to pull down β-catenin and E-cadherin in *Pmp22+/+* sciatic nerves. **(E)** Immunoprecipitation using control IgG and anti-β-catenin antibodies was carried out using extracts from mouse sciatic nerves at P10, 3 and 6 month-old *Pmp22+/+* mice. The presence of E-cadherin, σ-catenin, or PAK1 in this IP was evaluated by immunoblotting. The β-catenin antibodies were able to pull down E-cadherin, σ-catenin and PAK1 in *Pmp22+/+* sciatic nerves, but β-catenin failed to pull down PAK1 in *Pak1-/-* nerves. **(F1)** Schwann cells were transfected with σ-catenin siRNA for 72 hours. Endogenous σ-catenin and T212 were evaluated by immunoblotting. β-Tubulin was used as loading control. **(F2)** The σ-catenin level was normalized against β-Tubulin level. T212 level was normalized against t-PAK1 level. Quantitative analysis showed an 85% knockdown of σ-catenin level, compared to that in control siRNA. The levels of T212 were significantly increased in the σ-catenin-siRNA cells. ** *P* < 0.01, *** *P* < 0.001. **(G1)** Western blot analysis of T212 and PAK1 were performed in Schwann cells following transfection of β-catenin and control siRNAs. **(G2)** Quantitative analysis shows a 95% knockdown of the β-catenin level. The levels of T212 were significantly increased in the β-catenin-siRNA cells. *** *P* < 0.001.

Both T212 and S144 antibodies failed to stain mouse nerves in our immunofluorescence experiments. To determine whether PAK1 is associated with junction protein complexes, we first performed co-immunoprecipitation (co-IP) in mouse sciatic nerve lysates and verified interactions between E-cadherin, β-catenin and σ-catenin (protein elements of adherens junction) (Fig 6C, 6D and 6E) [22]. Next, by using β-catenin antibodies, we were able to pull down PAK1 from the sciatic nerve lysates (Fig 6E). Because β-catenin is known to reside in the non-compact myelin regions and is an element of myelin adherens junction complex [22], PAK1 would associate with adherens junction protein complex. This finding is also consistent with a previously published study showing interactions between PAK1 and β-catenin [23].

To understand how PAK1 is activated in *Pmp22*+/- myelin, we tested whether the disruption of adherens junction protein complex activates PAK1. We first verified the presence of adherens junction protein complex in culture Schwann cells, including β-catenin, σ-catenin and E-cadherin (S4 Fig). By using siRNA, β-catenin or σ-catenin was knocked down in culture Schwann cells. PAK1 activity indexed by T212 was increased in either β-catenin or σ-catenin knock-down cells compared with that in control cells treated with scramble siRNA (Fig 6F1, 6F2, 6G1 and 6G2). Together, these findings suggest that PAK1 is present in the junction protein complexes. PAK1 may be activated when the junction protein complex is not formed normally.

To determine how PMP22 deficiency affects adherens junctions, we speculated an interaction between PMP22 and E-cadherin. This speculation was based on our previous study showing interactions between PMP22 and other junction proteins with Ig or Ig-like extracellular domains [5]. HA- or GFP-tagged PMP22 and E-cadherin were co-expressed in 293a cells. The co-IP showed an interaction between PMP22 and the extracellular domain of E-cadherin (Fig 6A and 6B). When co-IP was done in mouse sciatic nerve lysates, interactions between endogenous PMP22 and E-cadherin were only detectable in mice younger than postnatal day 15 but not in adult nerves (Fig 6C). This is in agreement with our immunostaining showing that PMP22 was localized into non-compact myelin regions in developing nerves but confined to internodal compact myelin and separated from myelin junctions in adult nerves [5]. These findings suggest that PMP22 might affect junction protein complex formation through its interactions with junction proteins during development. Abnormally formed junction complex would thereby activate PAK1.

### Therapeutic effect of PAK1 inhibitor in *Pmp22*+/- mice

Heterozygous deletion of *PMP22* in patients with HNPP still leaves an intact allele of *PMP22*. The allele of *PMP22* results in a partial production of PMP22 proteins [8], which would allow a portion of normal myelin junctions formed. We reasoned that the activated PAK1 would further disrupt those normally formed junctions, presumably via alterations of actin polymerization. PF3758309 is a commercially available PAK inhibitor. Chow et al have tested this compound (25mg/kg) in a skin cancer mouse model with a **7–10 day** course of intraperitoneal (i.p.) injection [24]. The compound penetrated into the nervous system [25].

**Fig 7:**
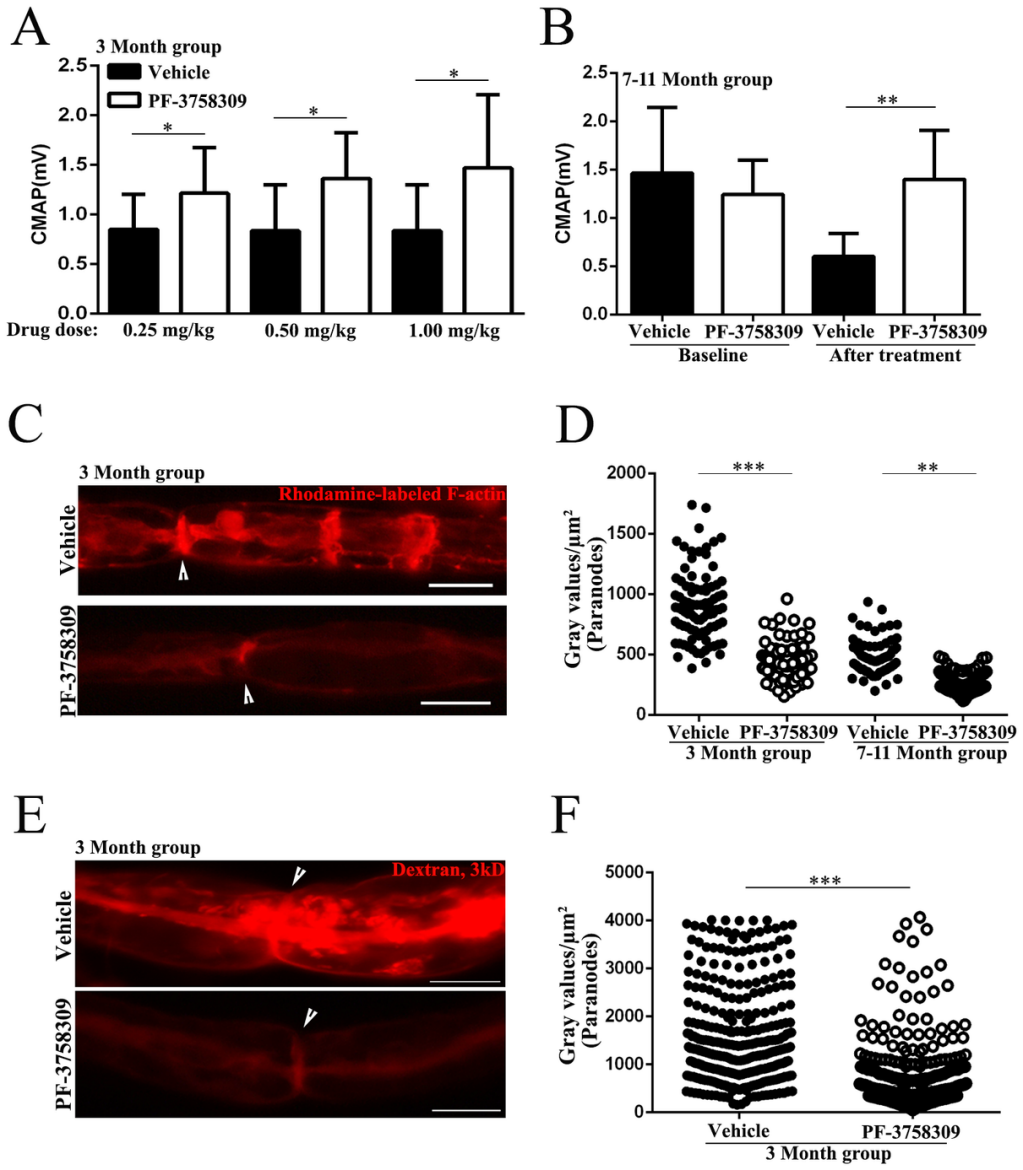
PAK1 inhibitor is therapeutic in *Pmp22+/-* mice. **(A)** NCS on mouse sciatic nerves showed significantly higher CMAP amplitude in 3-month-old *Pmp22+/-* mice treated with 0.25–1.0 mg/kg PF-3758309 (n = 42) for 11 weeks, compared with that in the vehicle group (n = 24). There was a trend of dose-dependent change. *P < 0.05. **(B)** This difference of CMAP amplitudes was also found between 7-12-month-old *Pmp22+/-* mice treated with 0.25 mg/kg PF-3758309 and the vehicle group. CMAP was measured every 10 days. By the end of one month, CMAP amplitudes were already significantly different between the treated and vehicle groups. Thus, the treatment was stopped at this point. The baseline CMAP amplitudes prior to the treatment were not different between the two groups but decreased over the course of treatment in the vehicle group and unchanged in the PF-3758309 group. ** *P* < 0.01. **(C)** Teased nerve fibers from 3-month-old *Pmp22+/-* mice were stained with the fluorescence-phalloidin to reveal F-actin. A nerve fiber from a PF-3758309-treated mouse showed a lower intensity of F-actin fluorescence when compared with that in a nerve fiber from a vehicle-treated mouse. Arrowheads point to the node of Ranvier, which are flanked by paranodes on each side. Scale bars = 10μm. **(D)** Fluorescence intensity of F-actin staining was quantified by placing 2.5μm x 2.5μm interest box 10μm away from the node of Ranvier. The intensity was compared between the PF-3758309 treated group and the vehicle group (n = 65–92 analyzed paranodes from 3 vehicle mice and 3 PF-3758309 treated mice). Note that a high level of F-actin in 3-month-old mice correlates well with a high level of PAK1 activity in the mice at the same age (Fig 5A). ** *P* < 0.01, *** *P* < 0.001. **(E)** Sciatic nerve fascicles from 3-month-old *Pmp22+/-* mice were incubated with 3kDa Dextran, as described [5], to evaluate the myelin permeability. Individual teased nerve fibers were imaged. Arrowheads point to the node of Ranvier. Notice that a nerve fiber from a mouse treated with vehicle showed higher fluorescence intensity than that in a nerve fiber from a mouse treated with PF-3758309. Scale bars = 10μm. **(F)** Fluorescence intensity was quantified by placing a 2.5μm x 2.5μm interest box 10μm away from the middle point of the node of Ranvier. The intensity was significantly decreased in 3-month-old PF-3758309-treated nerve fibers, compared to those from the vehicle group (n = 495–521 analyzed paranodes from 3 vehicle mice and 3 PF-3758309-treated mice). *** *P* < 0.001.

We first tested mouse tolerance to PF3758309 (i.p. daily). The dose of 2.5mg/kg or 25mg/kg killed over 50% of 20 *Pmp22*+/- mice within 15 days with drastic reduction of body weight, but no death was found in 21 vehicle-treated *Pmp22*+/- mice. Thus, *Pmp22*+/- mice were treated with 0.25, 0.5, 1.0mg/kg PF3758309 or vehicle—saline (Fig 7 and Table 1). Animals tolerated these lower doses well with no change of body weight or increase of death. To give a sufficient time for recovery of myelin permeability, we injected the compound for **11 weeks**. Injection started at age of postnatal day 7. The treatment prevented the decline of CMAP amplitudes with all three dosages (Fig 7A), but did not restore the CMAP amplitudes to the levels in *Pmp22*+/+ mice (3.2±0.6mV in ten 3-month-old *Pmp22*+/+ mice versus 1.5±0.8mV in eight 3-month-old *Pmp22*+/- mice treated with 1.00mg/kg PF-3758309). The remaining outcomes were collected only from mice treated with the lowest dose of 0.25mg/kg. PF3758309 suppressed levels of T212, phosphorylated MEK1 (S298) and F-actin (Table 1, Fig 7C and 7D), as well as improved abnormal claudin-19 distributions (i.e., tight junctions; Table 1) and myelin permeability (Fig 7E and 7F) compared with those in the vehicle group. Using teased nerve fibers, we quantified, as described [10], the percentages of nerve fibers with tomacula, a key pathology of HNPP. Tomacula were fewer in the treated group (Table 1).

**Table 1:** PAK1 inhibitor PF-3758309 is therapeutic in the *Pmp22+/-* mice

1 week old at start of injection (prior to tomacula formation)								
	mouse number	drug dose	CV(m/s)	Ratio (T212/ PAK1)	% Tomacula	% abnormal Claudin-19		Ratio (S298/ MEK1)
Vehicle	n = 8 (4F/4M)	0.25 mg/kg/day; via I.P.; duration = 11 weeks	20.3±1.5	0.75±0.0	35.8±5.5	55.7±16.6 ^a^	39.5±4.5 ^b^	0.90±0.1
PF-3758309	n = 8 (3F/5M)		17.8±3.0	0.61±0.0	27.3±4.5	36.5±18.4 ^a^	27.7±4.6 ^b^	0.39±0.2
*P* value			0.069	0.015	0.028	0.045	0.010	0.004
6–11 month age at start of injection (after tomacula reached their peak prevalence)								
	mouse number	drug dose	CV(m/s)	Ratio (T212/ PAK1)	%Tomacula + outlier	% Tomacula -outlier		% abnormal Claudin-19
Vehicle	n = 8 (3F/5M)	0.25 mg/kg/day; via I.P.; duration = 4 weeks ^c^	24.6±3.4	0.34±0.1	29.1±3.0	29.1±3.0		66.3±8.7 ^a^
PF-3758309	n = 8 (4F/4M)		19.7±11.1	0.26±0.1	25.2±7.5	22.8±4.3 ^d^		43.5±13.3 ^a^
*P* value			0.628	0.037	0.228	0.010		0.012

^a^ Quantification of paranodes with abnormal claudin-19 staining.

^b^ Quantification of incisures with abnormal claudin-19 staining.

^c^ At the 4th week of injection, NCS already detected a significant difference. Thus, the treatment was terminated earlier than 12 weeks for this group of mice.

^d^ An outlier was taken off from the PF-3758309 group.

It is important to determine whether the treatment is still effective after the developmental stage. Moreover, in human clinical trials, a range of ages, instead of a single age point, are usually included. Mice were enrolled at ages of 6–11 months. Again, F-actin levels were significantly lower in the PF3758309 (0.25mg/kg) group (Fig 7D). In Fig 7B, we measured CMAP amplitudes prior to the treatment. After treatment, CMAP amplitudes decreased about a half (from 1.6±0.7mV to 0.7±0.2mV) in the vehicle group, but the decrease was **completely** prevented over the course of PF3758309 treatment. The measurement of baseline CMAP was not possible in the 1^st^ set of experiment since the injection started at age of 1 week when mouse paws were too small for any reliable recording.

The percentages of tomacula in the 2^nd^ set of experiment were not significantly different between PF3758309 and vehicle groups. However, we noticed one outlier with the highest percentage of tomacula (39.7%) in PF3758309 group. We then counted another 14 *Pmp22*+/- mice (un-injected). None of them showed tomacula above 39.7%. When the outlier was removed, the difference of tomacula between PF3758309 and vehicle group was significant (the 6-7^th^ column in Table 1). Finally, by Western blot, PAK1 activity indexed by T212 was decreased in PF3758309 groups compared with vehicle groups. The claudin-19 distribution was also improved in the treated group (Table 1).

Two additional PAK inhibitors (FRAX597 and FRAX486) were commercially available. However, they failed to inhibit PAK1 activity in mouse peripheral nerves (Ratio of T212/PAK1: vehicle 1.07± 0.23 versus FRAX597 1.30±0.62, n = 4 vehicle mice and 5 FRAX597 mice, p = 0.502; vehicle 0.97± 0.13 versus FRAX486 0.93±0.13, n = 4 vehicle mice and 5 FRAX486 mice, p = 0.601). They were not suitable for the treatment. This failure was presumably due to the poor penetrance into the peripheral nerve system.

### PAK inhibitor suppresses F-actin formation via PAK1

**Fig 8:**
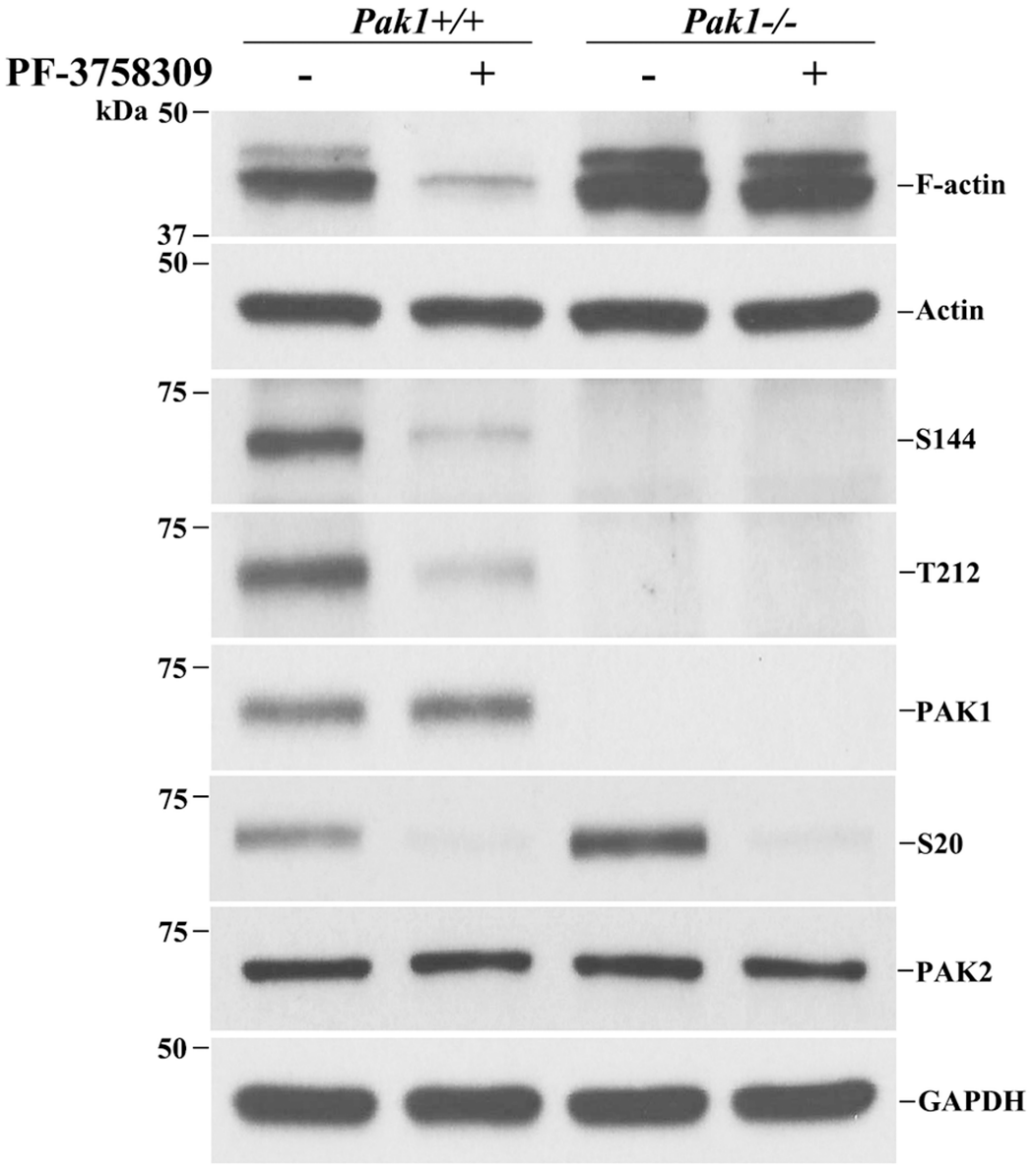
PAK inhibitor PF-3758309 blocks actin polymerization via PAK1. F-actin, phosphorylated PAK1 (S144, T212) and phosphorylated PAK2 (S20) were analyzed in *Pak1+/+* and *Pak1-/-* primary Schwann cell culture after the cells were treated with PF-3758309 (9μM) for 12 hour. PF-3758309 suppressed the levels of F-actin in *Pak1+/+* Schwann cells but failed to do so in *Pak1-/-* Schwann cells. The total actin and GAPDH were used as loading controls. Note that the specificity of S20 antibody has been demonstrated by Zhan et al [42].

Suppression of F-actin formation by PF3758309 may not be due to PAK1 but an off-target effect. One may test this issue by crossing *Pak1*-/- into *Pmp22*+/- mice to remove *Pak1* gene in *Pmp22*+/- mice. However, a compensation effect of PAK2 activity in *Pak1*-/- mice has been observed [26]. We have confirmed an increase of phosphorylated PAK2 (S20) in *Pak1-/-* Schwann cells (row 6 in Fig 8). Thus, this approach does not help.

When Schwann cells were treated with either vehicle or PF3758309, F-actin became hardly detectable in *Pak1*+/+ cells. However, PF3758309 failed to change the levels of F-actin in *Pak1*-/- cells (row 1 in Fig 8). This finding suggests that PF3758309 suppresses F-actin formation mainly via PAK1.

Note that F-actin formation may be regulated by many different signaling pathways, not just PAK1. Thus, deletion of PAK1 alone in cells does not necessarily decrease the level of F-actin, which could be even increased by unidentified compensating mechanisms, such as PAK2 (row 1 in Fig 8). This notion was in line with the fact that PF3758309 inhibited all levels of F-actin, phosphorylated PAK1 (T212 in Table 1) and phosphorylated PAK2 (S20) (row 6 in Fig 8). This also suggests that PF3758309 is not a specific inhibitor for PAK1, but affects other PAKs.

## Discussion

Our study revealed a novel mechanism—myelin junctions are disrupted through PAK1 activity in HNPP mouse model. This change results in conduction block in the *Pmp22*+/- nerves, thereby explaining focal sensory/motor deficits in HNPP. Moreover, myelin junction disruption occurred much earlier than segmental demyelination in *Pmp22*+/- mice [5, 10, 11]. Therefore, functional demyelination represents an upstream mechanism prior to the actual stripping of myelin, a key pathology shared by many demyelinating diseases. We believe that this is an important conceptual advance since this mechanism reveals molecular targets that may be intervened prior to the removal of myelin.

The conclusion above is supported by several lines of evidence. First, PAK1 activity indexed by T212 is increased in *Pmp22*+/- nerves. The time course of the PAK1-increase mirrors the progression of tomacula [10]. Second, based on our co-IP data, PAK1 interacts with adherens junction protein complexes either directly or indirectly, as demonstrated previously [23]. Interactions between PMP22 and other myelin-junction-related proteins have also been found in our previous study [5]. Because all myelin junctions are localized to the non-compact myelin regions [5], activated PAK1 would be available to affect other types of junctions and F-actin. T212 phosphorylation in PAK1 has been shown to recruit PAK1 to submembrane actins [20], where PAK1 activity may be further promoted by certain lipids, such as sphingosine or phosphoinositides, independent of small GTPases [27, 28]. Thus, an increase of T212 in *Pmp22*+/- nerves is highly relevant for F-actin formation locally around the myelin junctions.

This finding is consistent with numerous studies in epithelial cells that demonstrated junction disruption after altering actin polymerization [29, 30]. This finding is also in line with another study in transected mouse sciatic nerves; an increase of F-actin in myelin through the activation of a small GTPase, Rac1, promoted the removal of E-cadherin, a marker of adherens junctions. Inhibition of actin polymerization prevented the E-cadherin from being removed [16].

Interestingly, septate junctions are spared in *Pmp22*+/- nerves [5, 10], which was replicated in the present study. Like epithelial cells, myelinating Schwann cell polarizes into “apical-like and basolateral-like” domains [31]. We speculate that septate junctions are involved in a domain and mechanisms distinct from other myelin junctions. Indeed, in our previous study [5], PMP22 was found in the paranodal regions during the early development but was not observed in the septate junction region.

Third, PAK inhibitor (PF3758309) improved F-actin dysregulation, junction disruption, and abnormal myelin permeability in *Pmp22*+/- nerves. The decline of CMAP amplitudes was completely prevented by the PAK inhibitor. The inhibitor was even effective in the adult nerves with fully developed pathology (Fig 7 and Table 1). These results were from a large cohort of mice (total n = 82 mice) of two age groups with three different dosages. Effective dose of PF3758309 (0.25mg/kg) was **100 times lower** than the dose of 25mg/kg used in skin cancer mouse model [24]. Such low dose of 0.25mg/kg PF3758309 makes its use safer. Indeed, we treated the mice **for 11 weeks**, which was a long duration rarely seen in literature and far longer than the duration of 7–10 days used in the skin cancer mouse model [24]. Yet, there was no increase of mouse mortality and observable side-effect.

PAK inhibitor was effective in aged animals (6–11 months; Table 1). While pharmacological treatment is unlikely given to asymptomatic patients, the PAK inhibitor would still be effective in the symptomatic patients with the pathology fully developed. This is also in line with our previous observation of dynamic paranodal changes during action potential propagation in adult nerves [32]. Thus, PAK inhibitors may become a promising therapy for HNPP.

Our data suggest that PF-3758309 also inhibits PAK2 in the peripheral nerves. Thus, the beneficial effect in the treated *Pmp22*+/- mice could relate to other types of PAKs, in addition to PAK1. Loss-of-function of PAK3 has been shown to affect synaptic plasticity [33]. Although the effective dose of PF-3758309 was very low in our study, it remains to be determined whether the treatment results in any side-effect in cognitive functions.

The severity of myelin permeability varied in different *Pmp22*+/- nerve fibers (Fig 1B in Guo et al Ann Neurol 2014) [5]. This variability would produce two different populations of myelinated nerve fibers in HNPP. Those in the first group have severely "leaky" myelin (i.e., high capacitance), leading to failure of action potential propagation in the absence of demyelination (Fig 3). We call this "functional demyelination". Inhibition of PAK1 is expected to restore the nerve conduction in the group of nerve fibers, which is reflected by the prevention of CMAP decline in PF3758309-treated *Pmp22+/-* mice (Fig 7 and Table 1). Those in the second group have mildly increased permeability of myelin, which still allows action potentials to propagate but would partially compromise the safety factor of action potential propagation. This compromised safety factor would put the PMP22-deficient nerve fibers at risk to conduction failure if the fiber is challenged by external factors, such as mechanical stress. Indeed, our previous study has demonstrated that mechanical compression induced conduction block in *Pmp22*+/- nerves faster than that in *Pmp22*+/+ nerves [10]. It is not until the very late stage (>10–12 months) when obvious segmental demyelination and axonal loss start in *Pmp22*+/- mice [10, 34, 35]. Note conduction velocities in NCS are determined by large myelinated nerve fibers [1]. As long as there are some large myelinated nerve fibers still conducting action potentials in *Pmp22*+/- nerves, conduction velocities would remain normal or minimally decreased. This is also consistent with the observations in patients with HNPP [11].

**Fig 9:**
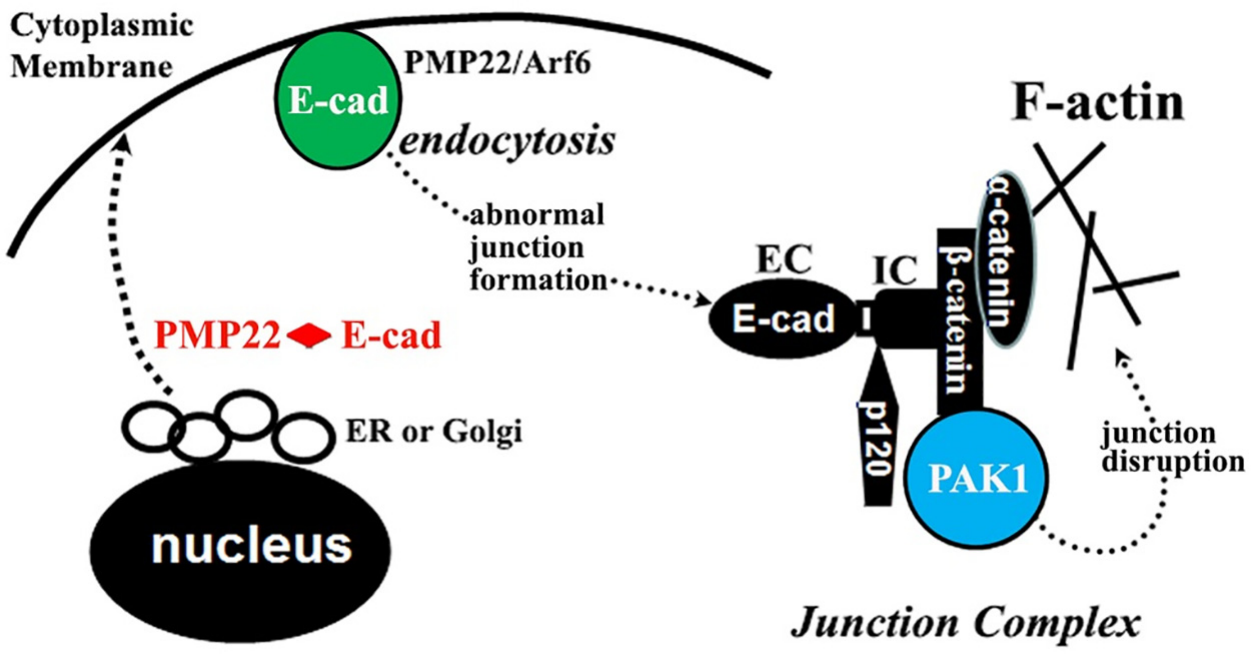
A proposed mechanism for junction disruption in HNPP. Initiation—PMP22 and E-cadheren travel via the secretory pathway from endoplasmic reticulum (ER)/Golgi apparatus to cytoplasmic membrane [36, 37]. PMP22 has been reported to regulate the endocytosis of E-cadheren via Arf6 (an ATPase) [38]. PMP22 may also form a protein complex with E-cadherin during the secretory pathway. Deficiency of PMP22 would affect the transport processes of E-cadherin or other junction proteins, thereby leading to the abnormal formation of junction complex. Perpetuation—β-catenin in adherens junction protein complex has been shown to interact with PAK1 [23]. Abnormal formation of junction complex activates PAK1 in *Pmp22*+/- Schwann cells (Fig 6B), which further promotes disruption of junction protein complexes. PAK1 is known to regulate actin polymerization [17]. Thus, activation of PAK1 may disrupt the junctions via actin polymerization.

In summary, we propose a pathogenic mechanism of two steps—initiation and perpetuation (Fig 9). Initiation: PMP22 and E-cadherin (or other junction proteins) travel via the secretory pathway from endoplasmic reticulum (ER)/Golgi apparatus to cytoplasmic membrane [36, 37]. Like the polarized epithelial cells, E-cadherin in developing Schwann cells has to be transported from apical domain (internodal membrane) to basolateral domain (paranode and incisures) through endocytosis [22, 37]. PMP22 has been reported to regulate the endocytosis of E-cadherin via Arf6, an ATPase [38]. PMP22 may affect E-cadherin transport during development (Fig 6). In supporting this notion, our study has shown that PMP22 is transiently expressed in non-compact myelin regions of developing nerves but disappears in those compartments after maturation [5]. Because abnormal junction formation is upstream to the PAK1 activation, the PAK inhibitor would not affect this step. Perpetuation: HNPP with heterozygous deletion of *PMP22* still have a normal allele that produces about a half of PMP22 proteins in normal controls [8]. These residual PMP22 proteins would permit some myelin junctions to form. However, after the activation of PAK1 by abnormally formed junction complex (Fig 6), PAK1 activity promotes further junction disruption in adulthood (Figs 2 and 6). Thus, this junction disruption, not the abnormal junction formation during early development, would be affected by the PAK inhibitor. It remains to be determined whether the junction disruption was through the increase of F-actin and/or via another unidentified pathway. However, studies in epithelial cells have demonstrated junction disruption by altering actin polymerization [29, 30]. After all, inhibition of PAK1 activity did suppress the formation of F-actin and improved claudin-19 distribution (Figs 7C, 7D and 8; Table 1). Together, these findings not only provide a mechanistic explanation for abnormal myelin permeability and impaired action potentials propagation in HNPP, but also offer a promising therapeutic approach for this disease.

## Materials and Methods

### Ethics statement

All mice were housed in Tennessee Valley Healthcare System (TVHS) animal facility, a part of Vanderbilt University animal care system. Experimental procedures were approved by the IACUC of Vanderbilt University (M1500006-00). All mice in this study were euthanized by CO2 asphyxiation, according to the guidelines of the IACUC at the Vanderbilt University and the recommendations of the Panel on Euthanasia of the American Veterinary Association.

### Animals and drug administration

*Pmp22+/-* mice were backcrossed with C57Bl6J mice (Jackson Lab) for more than 10 generations to reach congenic. Mice were genotyped as described [9]. The primers for genotyping are listed in S3 Table. The congenic *Pmp22*+/- mice have been extensively characterized [5]. They showed pathology and other features similar to those in *Pmp22*+/- mice with mixed background (C57Bl6J/129) [10].

*Pak1-/-* mice were from Dr. Jonathan Chernoff’s lab, Fox Chase Cancer Center, USA. The *Pak1*-/- mice were produced in C57Bl6J background and have been described with negligible phenotype [19].

For PAK1 inhibitor (PF3758309) injections, *Pmp22+/-* mice were randomized into vehicle and treated groups. Based on our power calculation of variations derived from mouse compound nerve action potentials, 7 mice for each group would have a 92% chance to detect a significant difference. PF-3758309 (Cat# CT-PF0375, ChemieTek) was dissolved in normal saline for intraperitoneal injection (i.p.) daily.

### Co-immunoprecipitation

Sciatic nerves or cells were lysed in immunoprecipitation buffer (Cat# 87788, Thermo scientific) with proteinase/phosphatase inhibitor cocktail and incubated with primary antibodies overnight at 4°C with rotation (70 rpm). Protein G agarose beads (Cat# 15920–010, Life technologies) were added for another 2 hour incubation at 4°C. Samples were eluted with Laemmli sample buffer (Cat# 161–0737, Bio-rad), resolved by SDS-PAGE, and analyzed by immunoblot. All antibodies and their titers in this study are listed in S4 Table.

### Evaluation of myelin permeability

This technique has been validated [5]. In brief, 1 cm sciatic nerve fascicles were submerged in artificial CSF after epineurium removal and sealed at both ends with Vaseline. A 3kDa Dextran of fluorescence (2 mg/ml, Cat# D3329, Life technologies) was added for one hour incubation at room temperature without oxygenation. After washing, the nerve fascicles were fixed in 4% PFA for 10 minutes and teased into individual nerve fibers on glass slides for fluorescence microscopy. Fluorescence intensity was quantified by placing a 5μm x 5μm interest box 10μm away from the middle point of the node of Ranvier.

### β-catenin knockdown in Schwann cells

Silencing of β-catenin and σ-catenin were carried out using Accell SMARTpool siRNAs (Cat# A-062106-13 and A-040628-15, Dharmacon). Schwann cells were transfected with 1μM siRNA. The efficiency of the knockdown was evaluated by Western blot 72 hours after the transfection. Accell non-targeting siRNA (Cat# D-001910, Dharmacon) was used as negative control.

### Immunofluorescence staining

This method was modified from our published study [5, 10]. In brief, sciatic nerves were fixed, embedded in paraffin, and cut into 5μm-thick slices. Sections were incubated overnight with primary antibodies at 4°C. After washing, sections were stained for 1 hour with secondary antibodies. The stained slides were examined under a Leica fluorescent microscope (Leica DM6000B). For teased nerve stains, sciatic nerves were fixed in 4% paraformaldehyde (PFA) overnight and teased into individual fibers on glass slides. The slides were dried overnight, reacted with primary antibodies, and followed by secondary antibodies. For newly formed F-actin staining, as described [16], the existing F-actin in sciatic nerve explants was saturated with a cell-permeable actin-binding compound, jasplakinolide (Cat# 420127, Millipore), at 1μM in culture media. After washing with PBS, the explants were incubated in a drug-free media for 6 hours at 37°C. The explants were fixed in 4% PFA and teased for F-actin staining using fluorescent phalloidin (1:400, Cat# R415, Life Technologies).

### Nerve conduction study (NCS)

NCS was previously described [10]. In brief, mice were anesthetized with isoflurane (VetEquip Inc. Cat# 908106; 1.7L/minute of oxygen at 1.0 bar; 1.5% of the total oxygen flow being vaporized with Isoflurane). This anesthetic drug has been tested in our laboratory. It does not affect nerve conduction if the procedure is completed within 25 minutes. A skilled technician in our lab gets NCS done in each mouse within 6 minutes. For the experiments of conduction block in Fig 3, Avertin (250mg/kg, i.p.) was used for anesthesia. Avertin did not affect CMAP over 2 hours [5]. CMAP was recorded from the intrinsic foot muscle using needle electrodes. Stimulation electrodes were positioned percutaneously at the sciatic notch and adjacent to the tibial nerve at the ankle. CMAP amplitudes were measured from baseline to the peak of negative deflection.

### Morphometric analysis of mouse sciatic nerves and electron microscopy with high pressure freezing

This method has been described [39]. Epon sections (1μM thickness) of mouse sciatic nerves were examined under the 63X objective. The entire field of transverse sections of each nerve was imaged for analysis. Images were imported into software (ImagePro Plus). Areas of each field were counted to obtain the number of nerve fibers.

Electron microscopy on mouse sciatic nerves was performed as described [40]. Briefly, sciatic nerves were cryofixed in a high-pressure freezer (HPM100; Leica) and freeze substitution was performed in an embedding system at low temperature (AFS; Leica) using the tannic acid–OsO_4_ protocol. Samples were embedded in Epon, sectioned (Ultracut S Ultramicrotome, Leica), and stained with an aqueous solution of 2% uranyl acetate followed by lead citrate. Samples were examined in a LEO EM 912AB electron microscope (Zeiss, Oberkochen, Germany). Pictures were taken with an on-axis 2048x2048-CCD-camera (TRS, Moorenweis, Germany).

### Western blot

Chopped sciatic nerves were immediately dropped into RIPA buffer (Cat# R0278, Sigma) with proteinase/phosphatase inhibiter cocktail (Cat# 5872, Cell Signaling). Samples were homogenized for protein isolation. Protein concentration was determined by BCA assay (Prod#23225, Thermo Scientific). Samples were loaded into SDS-PAGE gels and transferred to a PVDF membrane. The membranes were blotted with 5% non-fat milk and incubated overnight at 4°C with primary antibodies, and followed by secondary antibodies. The immune complexes were detected by the enhanced chemoilluminescence (Cat# NEL103001, Perkin Elmer). In some cases, the blots were stripped and re-probed with additional antibodies. Quantification of band intensity was performed by the ImageJ software (http://rsbweb.nih.gov/ij/).

### Plasmids and transfection

E-cadherin-GFP plasmid was purchased from Addgene (Cat# 28009). PMP22-HA was obtained from Genocopoia (Cat# EX-D0117-M06). The primers for E-cadherin pE-cad-(E+T)-GFP were, forward: 5’-CCCAAGCTTGCCACCATGGGCCCTTGGAGCCGC-3’, reverse: 5’-CCGCTCGAGAAACAGCAAGAGCAGCAGAATCAG-3’; pE-cad-(T+I)-GFP, forward: 5’-CGGGGTACCGCCACCATGATTCTGGGGATTCTTGGAGG-3’, reverse: 5’-CCGCTCGAGGTCGTCCTCGCCGCCT-3’. The accuracy of all plasmids was verified by DNA sequencing. The plasmids were transfected into HEK293a cells by using Effectene (Cat# 301425, Qiagen) according to the manufacturer’s instructions.

### Statistics

Statistical analysis was performed using GraphPad Prism software version 6.0 or SAS 9.4. The data was represented as the mean ± SD. For normally distributed data, a Student *t* test was utilized. The Wilcoxon Rank-sum test was used when the data were not under normal distribution. Differences were considered significant when the *P* value was less than 0.05.

## Supporting Information

S1 Fig Myelin junction abnormalities were observed under electron microscopy. Adult mouse sciatic nerves were imaged under electron microscopy after processed through high-pressure freezer and freeze substitution to ensure excellent preservation of paranodal myelin. (**A**) Tight junctions between paranodal loops were clearly visible in *Pmp22*+/+ nerves (arrowheads). (**B**) In contrast, there was only one tight junction visible in a *Pmp22*+/- paranode without a tomaculae. We cannot completely exclude that this change may be resulted from processing artifact. However, this preservation is the best that one could achieve with current technology. (**C**) In a different *Pmp22*+/- paranode with a tomaculae, large split spaces between myelin lamina were evident and devoid of any tight or adherens junctions. This abnormality was never observed in *Pmp22*+/+ nerves. Tight junctions and adherens junctions were not visible in these regions. As discussed in our previous publication [1], the paranodal lamina splitting might also be contributed by the loss of transmembrane adhesion molecules, such as JAM3 [2]. (**D**) This figure shows the tracing of all layers of EM tomography. The large paranodal lamina split involves all layers.
(TIF)

S2 Fig Localization of Caspr and Neurofascin in 3-month-old *Pmp22+/-* mouse nerves was not altered. (**A**) Longitudinal sections of 3-month-old *Pmp22*+/+ and *Pmp22+/-* mouse sciatic nerves were stained with antibodies against Caspr. Caspr immunoreactivity was normal in the paranodal (arrowhead) regions. (**B**) Teased never fibers of 3-month-old *Pmp22*+/+ and *Pmp22+/-* mouse sciatic nerves were stained with antibody against neurofascin. The pattern of neurofascin staining in *Pmp22+/-* paranodal (arrowhead) or incisures (arrow) was normal, even though the intensity of neurofascin appeared to be increased. (**C**) When comparison was made between the two genotypic groups, there was no significant difference of Caspr-stained paranodes from 3 months to 10 months of age (n = 55–110 paranodes and 310–440 incisures from either 3 *Pmp22+/+* or 3 *Pmp22+/-* mice at each age group; p>0.05). (**D**) The percentages of neurofascin-stained paranodes or incisures from 3 months to 10 months of age were also not different between the two genotypic groups (n = 240–350 paranodes and 800–1,100 incisures from 3 *Pmp22+/+* and 3 *Pmp22+/-* mice at each age group; p>0.05).
(TIF)

S3 Fig Ablation of *Pak1* in mice results in no phenotype in the peripheral nerves. (**A**) Genotyping detected *Pak1*+/+, *Pak1*+/- and *Pak1*-/- mice. (**B**) Duration on a rotating bar was tested using Rotarod. There was no significant difference between *Pak1*+/+ and *Pak1*-/- mice. (**C, D**) Nerve conduction studies were performed on the sciatic nerves and showed no significant difference of CMAP amplitude or conduction velocity between *Pak1*+/+ and *Pak1*-/- mice. (**E**) The mouse sciatic nerves were examined by semithin sections. There was no abnormality was found in *Pak1*+/+ and *Pak1*-/- mice up to 17 months of age.
(TIF)

S4 Fig Total PAK2 was not increased in *Pmp22*+/- nerve fibers. PAK2, but not PAK3, were detectable in mouse sciatic nerves by Western blot. The level of PAK2 was normal in *Pmp22*+/- nerves.
(TIF)

S5 Fig Characterization of adherens junction protein complex in conditionally immortalized mouse Schwann cells. Conditionally immortalized Schwann cells were generated as described [3]. The property of primary cells is largely preserved in these cells [3]. In brief, *Pmp22*+/+ or *Pak1*-/- mice were crossed with *SV40tg* transgenic mice to produce *Pmp22+/+/SV40tg* or *Pak1-/-/SV40tg* mice. At P5, sciatic nerves were dissected to culture Schwann cells at 33°C. The low temperature activated *SV40tg* transgene to express SV40 that promoted cell proliferation to a large quantity [2, 4]. Cells were allowed to differentiate by transferring them to a 37°C incubator, which inactivated the *SV40* transgene. *Pmp22*+/+/*SV40tg* Schwann cells exhibited their typical spindle shape. (**A**) These Schwann cells expressed a Schwann cell marker of S100. (**B**) To determine whether adherence junction protein complex is present in the Schwann cells, Co-IP was performed and showed that β-catenin was able to pull-down E-cadherin and P120.
(TIF)

S1 Table Nerve conduction studies in mouse sciatic nerves. *Data in S1 Table and S2 Table were collected from mice with mixed C57B6/129 background before the C57Bl6 congenic mice were available in our lab. CMAP amplitudes in the table above appear to be higher than those in congenic mice shown in Fig 7. This difference would not affect any conclusion since CMAP and the morphometric data in S2 Table were collected at the same time in the same mice.
(DOCX)

S2 Table Morphometric analysis in mouse sciatic nerves. *The total numbers of myelinated nerve fibers were not significantly different between *Pmp22*+/+ and *Pmp22*+/- mice. Because the transverse areas of sciatic nerves were normal in *Pmp22*+/- mice (for 3-month old mice, *Pmp22*+/+ 92,659±6,011μm^2^ versus *Pmp22*+/- mice 107,059±18,535μm^2^; p = 0.16), this comparison was equivalent to a comparison of nerve fiber density. In addition, all semithin sections of these sciatic nerves showed no signs of axonal degeneration, including axonal atrophy, accumulation of intra-axonal organelles and myelin collapsed into axons [5]. Note that the smallest myelinated nerve fibers on semithin sections may not be clearly visualized and accurately measured under light microscopy. They were omitted from the analysis. This would affect the total number of myelinated nerve fibers, but would not affect the conclusion since HNPP mainly affects the large diameter myelinated nerve fibers [6]. The abnormalities of myelin junctions in *Pmp22*+/- nerves are predominantly seen in large myelinated nerve fibers. **Because amplitudes of CMAP are mainly contributed by myelinated nerve fibers with large diameters [7], we also compared the number of nerve fibers with diameters ≥5μM. No significant difference was found between *Pmp22*+/+ and *Pmp22*+/- mice.
(DOCX)

S3 Table Mouse genotyping primers. (DOCX)

S4 Table Primary antibodies. * Claudin-19: gift from Dr. Furuse M. Kyoto University, Japan; JAM-C: gift from Professor Beat Imhof, CMU-University of Geneva, Switzerland; Pan Neurofascin: gift from Professor Peter Brophy, The University of Edinburgh, Scotland.
(DOCX)

S1 References Supporting References. (DOCX)
