# Supplementary figures and images for: Tuning PAK Activity to Rescue Abnormal Myelin Permeability in HNPP
Source: PLoS Genet. 2016 Sep 1;12(9):e1006290. doi: 10.1371/journal.pgen.1006290 (PMC5008806; doi:10.1371/journal.pgen.1006290)

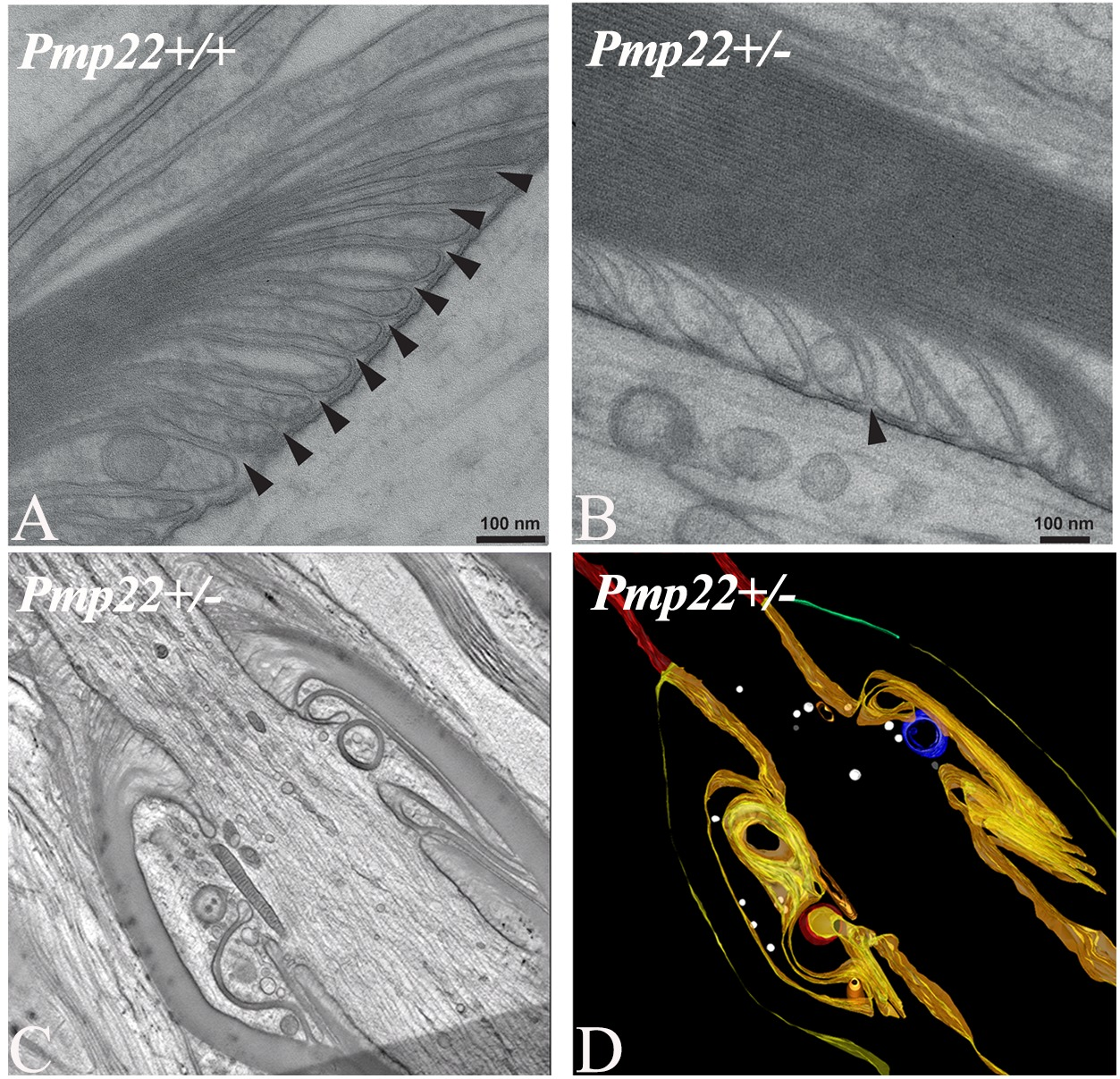

Supplement: S1 Fig — Adult mouse sciatic nerves were imaged under electron microscopy after processed through high-pressure freezer and freeze substitution to ensure excellent preservation of paranodal myelin. (A) Tight junctions between paranodal loops were clearly visible in Pmp22+/+ nerves (arrowheads). (B) In contrast, there was only one tight junction visible in a Pmp22+/- paranode without a tomaculae. We cannot completely exclude that this change may be resulted from processing artifact. However, this preservation is the best that one could achieve with current technology. (C) In a different Pmp22+/- paranode with a tomaculae, large split spaces between myelin lamina were evident and devoid of any tight or adherens junctions. This abnormality was never observed in Pmp22+/+ nerves. Tight junctions and adherens junctions were not visible in these regions. As discussed in our previous publication [1], the paranodal lamina splitting might also be contributed by the loss of transmembrane adhesion molecules, such as JAM3 [2]. (D) This figure shows the tracing of all layers of EM tomography. The large paranodal lamina split involves all layers. (TIF) [file pgen.1006290.s001.tif]

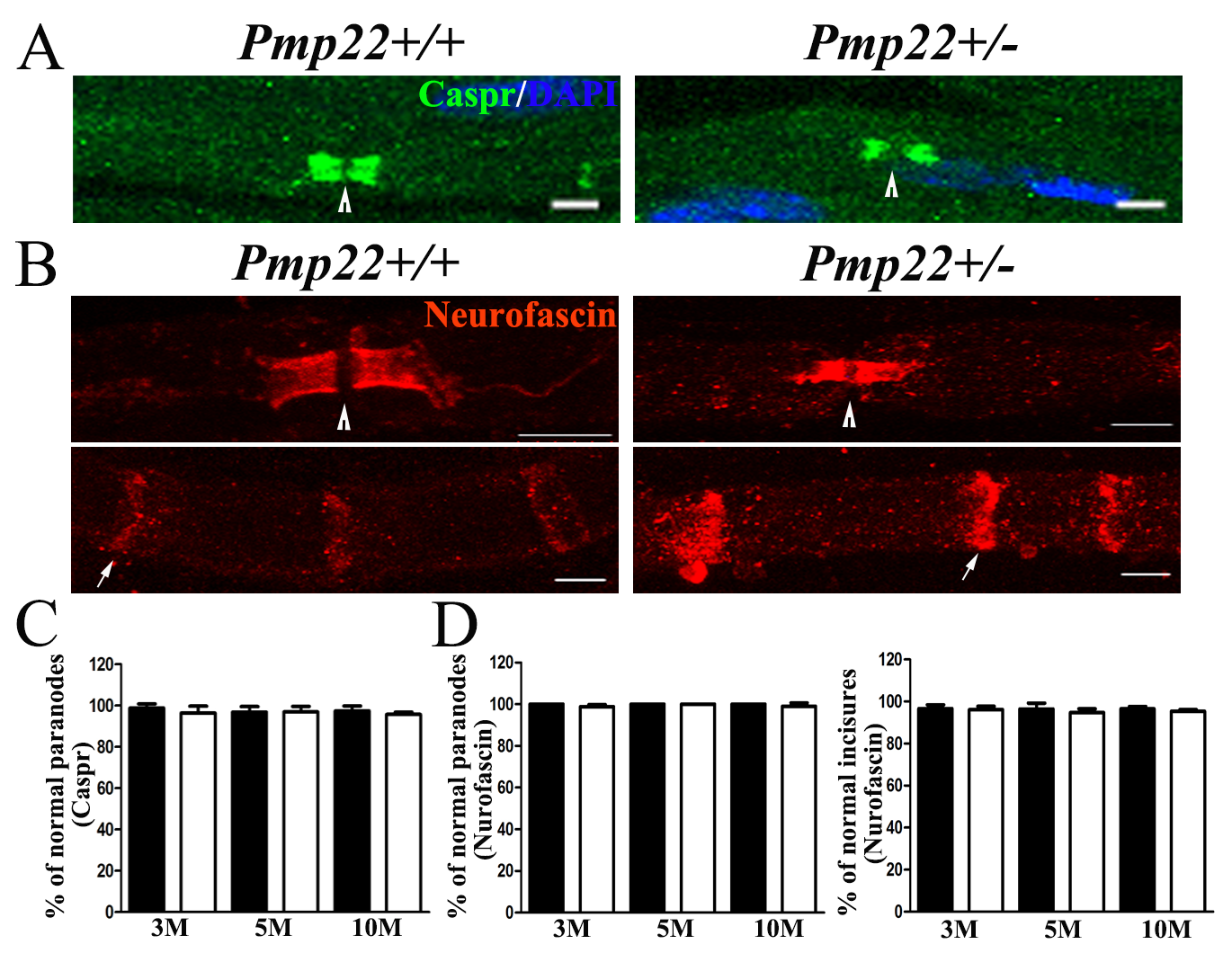

Supplement: S2 Fig — (A) Longitudinal sections of 3-month-old Pmp22+/+ and Pmp22+/- mouse sciatic nerves were stained with antibodies against Caspr. Caspr immunoreactivity was normal in the paranodal (arrowhead) regions. (B) Teased never fibers of 3-month-old Pmp22+/+ and Pmp22+/- mouse sciatic nerves were stained with antibody against neurofascin. The pattern of neurofascin staining in Pmp22+/- paranodal (arrowhead) or incisures (arrow) was normal, even though the intensity of neurofascin appeared to be increased. (C) When comparison was made between the two genotypic groups, there was no significant difference of Caspr-stained paranodes from 3 months to 10 months of age (n = 55–110 paranodes and 310–440 incisures from either 3 Pmp22+/+ or 3 Pmp22+/- mice at each age group; p>0.05). (D) The percentages of neurofascin-stained paranodes or incisures from 3 months to 10 months of age were also not different between the two genotypic groups (n = 240–350 paranodes and 800–1,100 incisures from 3 Pmp22+/+ and 3 Pmp22+/- mice at each age group; p>0.05). (TIF) [file pgen.1006290.s002.tif]

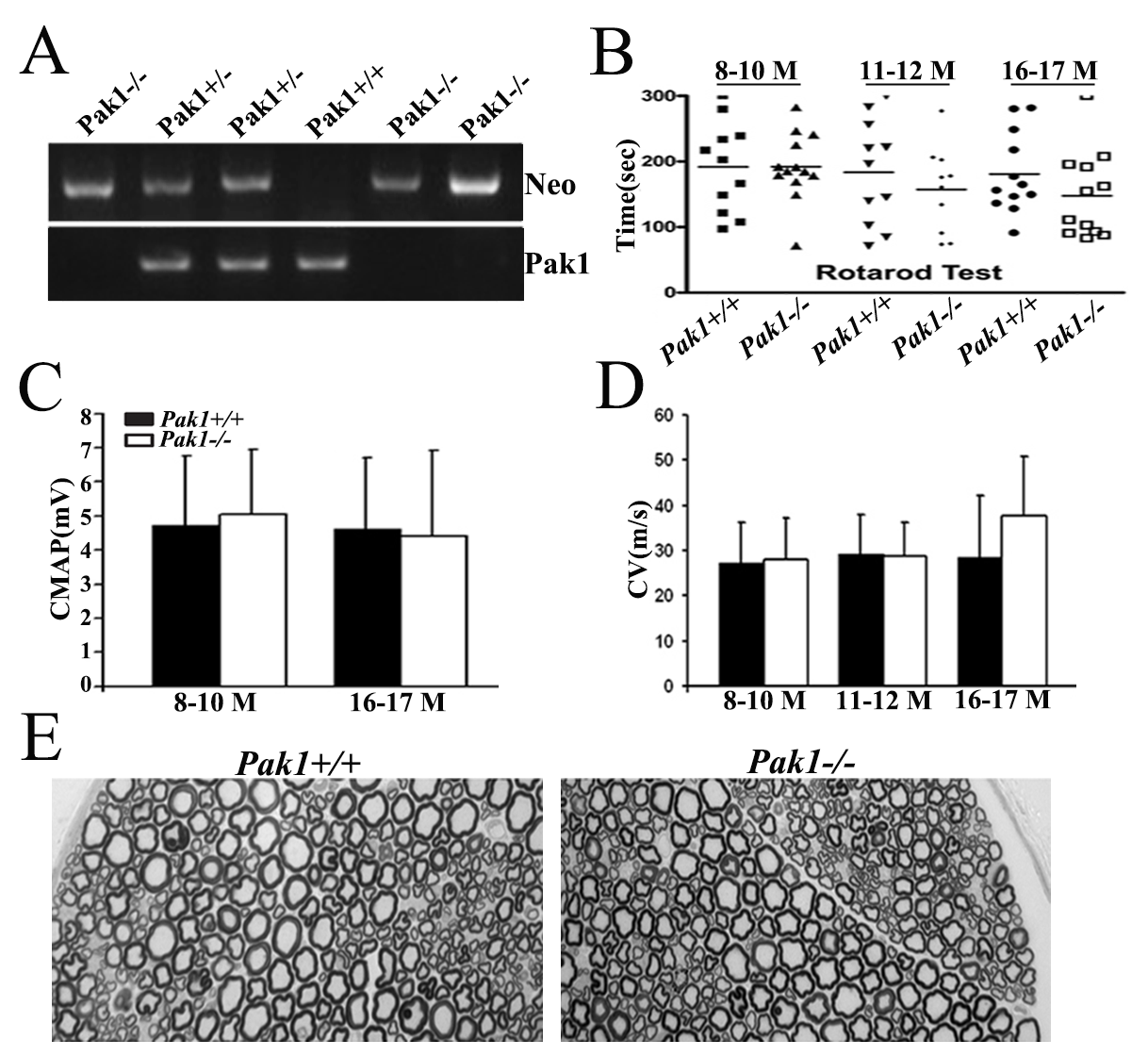

Supplement: S3 Fig — (A) Genotyping detected Pak1+/+, Pak1+/- and Pak1-/- mice. (B) Duration on a rotating bar was tested using Rotarod. There was no significant difference between Pak1+/+ and Pak1-/- mice. (C, D) Nerve conduction studies were performed on the sciatic nerves and showed no significant difference of CMAP amplitude or conduction velocity between Pak1+/+ and Pak1-/- mice. (E) The mouse sciatic nerves were examined by semithin sections. There was no abnormality was found in Pak1+/+ and Pak1-/- mice up to 17 months of age. (TIF) [file pgen.1006290.s003.tif]

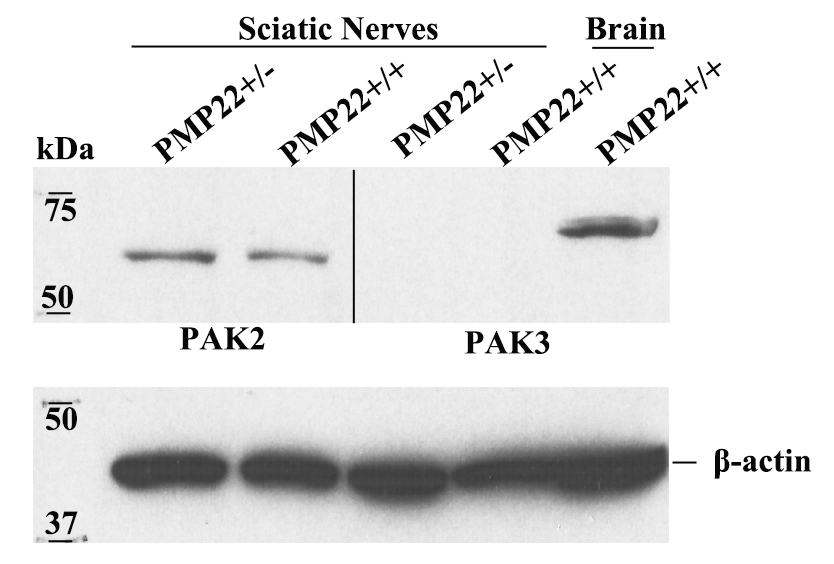

Supplement: S4 Fig — PAK2, but not PAK3, were detectable in mouse sciatic nerves by Western blot. The level of PAK2 was normal in Pmp22+/- nerves. (TIF) [file pgen.1006290.s004.tif]

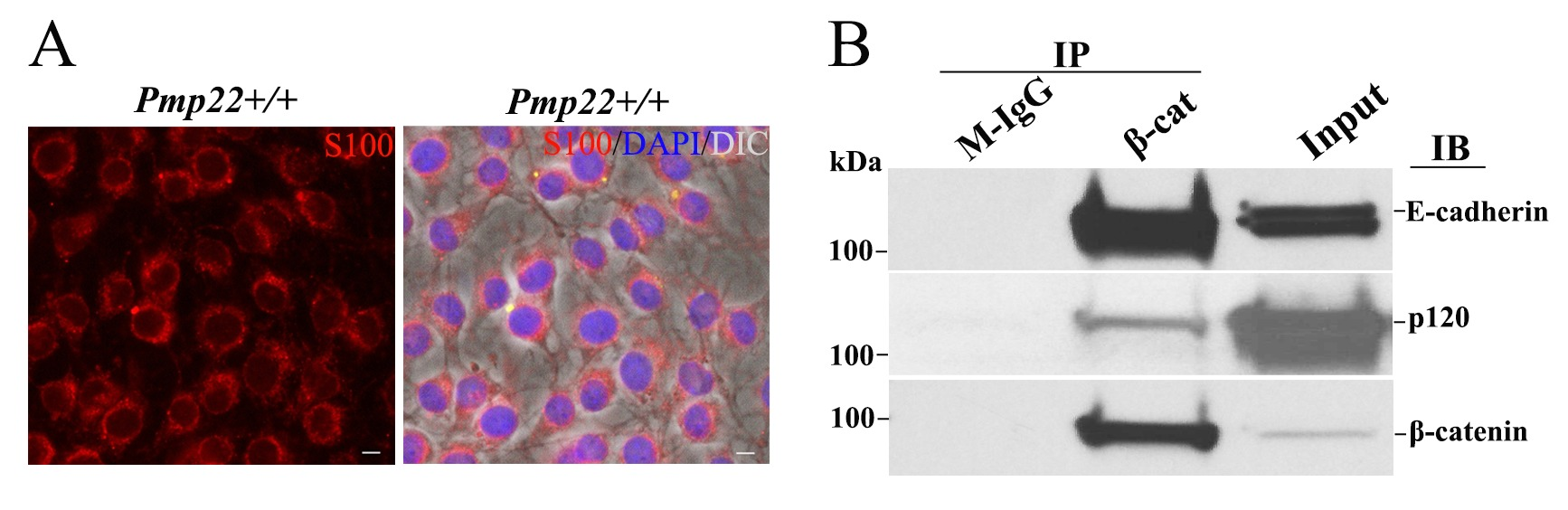

Supplement: S5 Fig — Conditionally immortalized Schwann cells were generated as described [3]. The property of primary cells is largely preserved in these cells [3]. In brief, Pmp22+/+ or Pak1-/- mice were crossed with SV40tg transgenic mice to produce Pmp22+/+/SV40tg or Pak1-/-/SV40tg mice. At P5, sciatic nerves were dissected to culture Schwann cells at 33°C. The low temperature activated SV40tg transgene to express SV40 that promoted cell proliferation to a large quantity [2, 4]. Cells were allowed to differentiate by transferring them to a 37°C incubator, which inactivated the SV40 transgene. Pmp22+/+/SV40tg Schwann cells exhibited their typical spindle shape. (A) These Schwann cells expressed a Schwann cell marker of S100. (B) To determine whether adherence junction protein complex is present in the Schwann cells, Co-IP was performed and showed that β-catenin was able to pull-down E-cadherin and P120. (TIF) [file pgen.1006290.s005.tif]
