# Supplementary material for: Tuning PAK Activity to Rescue Abnormal Myelin Permeability in HNPP
Source: PLoS Genet. 2016 Sep 1;12(9):e1006290. doi: 10.1371/journal.pgen.1006290 (PMC5008806; doi:10.1371/journal.pgen.1006290)
Supplement: S1 Table — *Data in S1 Table and S2 Table were collected from mice with mixed C57B6/129 background before the C57Bl6 congenic mice were available in our lab. CMAP amplitudes in the table above appear to be higher than those in congenic mice shown in Fig 7. This difference would not affect any conclusion since CMAP and the morphometric data in S2 Table were collected at the same time in the same mice. (DOCX) [file pgen.1006290.s006.docx]

| **S1 Table. Nerve conduction studies in mouse sciatic nerves** | | | | | | | |
| --- | --- | --- | --- | --- | --- | --- | --- |
|  | CMAP (mV)* | | |  | CV (m/s) | | |
|  | 2 Month age | 6 Month age | 12 Month age |  | 2 Month age | 6 Month age | 12 Month age |
| *Pmp22+/+* | 4.5±1.5 | 5.3±1.1 | 6.3±2.1 |  | 25.7±1.9 | 26.0±3.5 | 17.8±2.2 |
| *Pmp22+/-* | 2.2±1.0 | 2.1±0.8 | 3.0±0.7 |  | 25.7±2.0 | 23.0±3.3 | 17.3±5.6 |
| *P* value | <0.0001 | 0.0002 | 0.022 |  | 0.936 | 0.158 | 0.872 |
| n | 20 | 6 | 4 |  | 20 | 6 | 4 |
