# Supplementary material for: Tuning PAK Activity to Rescue Abnormal Myelin Permeability in HNPP
Source: PLoS Genet. 2016 Sep 1;12(9):e1006290. doi: 10.1371/journal.pgen.1006290 (PMC5008806; doi:10.1371/journal.pgen.1006290)
Supplement: S2 Table — *The total numbers of myelinated nerve fibers were not significantly different between Pmp22+/+ and Pmp22+/- mice. Because the transverse areas of sciatic nerves were normal in Pmp22+/- mice (for 3-month old mice, Pmp22+/+ 92,659±6,011μm2 versus Pmp22+/- mice 107,059±18,535μm2; p = 0.16), this comparison was equivalent to a comparison of nerve fiber density. In addition, all semithin sections of these sciatic nerves showed no signs of axonal degeneration, including axonal atrophy, accumulation of intra-axonal organelles and myelin collapsed into axons [5]. Note that the smallest myelinated nerve fibers on semithin sections may not be clearly visualized and accurately measured under light microscopy. They were omitted from the analysis. This would affect the total number of myelinated nerve fibers, but would not affect the conclusion since HNPP mainly affects the large diameter myelinated nerve fibers [6]. The abnormalities of myelin junctions in Pmp22+/- nerves are predominantly seen in large myelinated nerve fibers. **Because amplitudes of CMAP are mainly contributed by myelinated nerve fibers with large diameters [7], we also compared the number of nerve fibers with diameters ≥5μM. No significant difference was found between Pmp22+/+ and Pmp22+/- mice. (DOCX) [file pgen.1006290.s007.docx]

| **S2 Table. Morphometric analysis in mouse sciatic nerves** | | | | | | | | |  |
| --- | --- | --- | --- | --- | --- | --- | --- | --- | --- |
|  | 1 Months | |  | 3 Months | |  | 6 Months | |  |
|  | total fiber^*^ | ≥5µm fiber^**^ |  | total fiber | ≥5µm fiber |  | total fiber | ≥5µm fiber |  |
| *Pmp22+/+* | 34144±2151 | 15679±899 |  | 28939±3799 | 16573±2813 |  | 21579±1888 | 16598±1020 |  |
| *Pmp22+/-* | 30961±2993 | 15912±1318 |  | 25340±3976 | 18841±3634 |  | 18273±1597 | 15064±1242 |  |
| *P* value | > 0.05 | > 0.05 |  | > 0.05 | > 0.05 |  | > 0.05 | > 0.05 |  |
| n | 6 *Pmp22+/+*, 6 *Pmp22+/-* | |  | 9 *(Pmp22+/+)*, 5 *(Pmp22+/-)* | |  | 4 *(Pmp22+/+)*, 4 *(Pmp22+/-)* | |  |
