# Supplementary material for: Tuning PAK Activity to Rescue Abnormal Myelin Permeability in HNPP
Source: PLoS Genet. 2016 Sep 1;12(9):e1006290. doi: 10.1371/journal.pgen.1006290 (PMC5008806; doi:10.1371/journal.pgen.1006290)
Supplement: S3 Table — (DOCX) [file pgen.1006290.s008.docx]

| **S3 Table. Mouse genotyping primers** | | | | |
| --- | --- | --- | --- | --- |
| Gene | Prime | Sequences (5'-3') | Wt (bp) | KO/Tg (bp) |
| *Pmp22* | *Pmp22*-forward | CAGCCACCATGCTCCTACTC | 317 | 405 |
|  | *Pmp22*-reverse | CAGCCCTTGCTCACTGTCTAC |  |  |
|  | *Neo*-reverse | GCAGCGCATCGCCTTCTATC |  |  |
| *Pak1* | *Pak1*-forward | GCCCTTCACAGGAGCTTAATGA | 240 | 360 |
|  | *Pak1*-reverse | GAAAGGACTGAATCTAATAGCA |  |  |
|  | *Neo*-reverse | CATTTGTCACGTCCTGCACGA |  |  |
