# Supplementary material for: Tuning PAK Activity to Rescue Abnormal Myelin Permeability in HNPP
Source: PLoS Genet. 2016 Sep 1;12(9):e1006290. doi: 10.1371/journal.pgen.1006290 (PMC5008806; doi:10.1371/journal.pgen.1006290)
Supplement: S4 Table — * Claudin-19: gift from Dr. Furuse M. Kyoto University, Japan; JAM-C: gift from Professor Beat Imhof, CMU-University of Geneva, Switzerland; Pan Neurofascin: gift from Professor Peter Brophy, The University of Edinburgh, Scotland. (DOCX) [file pgen.1006290.s009.docx]

**S4 Table. Primary antibodies**:

| **Antibody** | **Source** | **Species raised** | **Specific antigen** | **Type** | **Reference** |
| --- | --- | --- | --- | --- | --- |
| F-actin | Abcam  #ab205 | mouse | NH3 recognises human Filamentous actin (F-actin) | Monoclonal IgM | 1 |
| GAPDH | Abcam  #ab9485 | rabbit | Full length native protein from human erythrocytes. | Polyclonal IgG | 2 |
| HA | Abcam  #ab9110 | rabbit | YPYDVPDYA(influenza hemagglutinin-HA-epitope) conjugated to KLH. | Polyclonal IgG | 3 |
| Actin | Millipore  #MAB1501 | mouse | Synthetic peptide corresponding to the N-terminal two thirds of the actin molecule, possibly near amino acids 50-70. | Monoclonal IgG1k | 4 |
| Claudin-19 | Dr Furuse M* | rabbit | C-terminal 19 amino acids of mouse claudin-19 | Polyclonal IgG | 5 |
| Rac1 | Millipore  #05-389 | mouse | Recombinant protein containing the full length human Rac | Monoclonal IgG2b | 6 |
| PAK1 | Cell Signaling  #2602 | rabbit | Synthetic peptide corresponding to the amino-terminus of human PAK1 | Polyclonal | 7 |
| PAK2 | Cell Signaling  #2608 | rabbit | Synthetic peptide corresponding to the amino-terminus of human PAK2 | Polyclonal | 8 |
| PAK3 | Cell Signaling  #2609 | rabbit | Synthetic peptide corresponding to the amino-terminus of human PAK3 | Polyclonal | 9 |
| Phospho-PAK1 (Ser144)/PAK2 (Ser141) | Cell Signaling  #2606 | rabbit | Synthetic phosphopeptide corresponding to residues surrounding Ser144 of human PAK1 | Polyclonal | 10 |
| β-actin | Abcam  # ab8227 | rabbit | Synthetic peptide of residues 1 - 100 of human beta Actin | Polyclonal IgG | 11 |
| Active Rac1 | NewEast Biosciences 26903 | mouse | Recombinant protein containing the full length active Rac | Monoclonal IgM | 12 |
| β-Tubulin | Cell Signaling  #2128 | rabbit | Synthetic peptide corresponding to the amino terminus of human β-tubulin. | Polyclonal IgG | 13 |
| E-Cadherin | BD Transduction Laboratories #610181 | mouse | Synthetic peptide corresponding to the sequence surrounding Pro780 of human E-cadherin protein. | Monoclonal IgG_2a_ | 14 |
| β-Catenin | BD Transduction Laboratories #610154 | mouse | Recombinant peptide of amino acids 571-781 of Mouse β-Catenin | Monoclonal IgG_1_ | 15 |
| PMP22 | AssaybioTech  #C0306 | rabbit | Synthesized peptide from C-terminal of human PMP22 | Polyclonal IgG | 16 |
| MAG | Abcam  #ab89780 | mouse | Corresponding to amino acids 119-208 of Human MAG with a proprietary tag | Monoclonal IgG1 | 17 |
| P120 | BD Transduction Laboratories #610134 | mouse | Recombinant peptide of amino acids 326-632 of Mouse p120 | Monoclonal IgG_1_ | 18 |
| GFP | GenScript *#*A01704 | rabbit | Recombinant full-length GFP protein | Polyclonal IgG | GenScript website |
| PAK1 (Phospho-Thr212) | Genway Biotech #GWB-961E2C | rabbit | Synthetic peptide corresponding to phosphorylation site of threonine 212 (P-V-T(p)-P-T) derived from human PAK1. | Polyclonal IgG | 19 |
| E-Cadherin | Cell Signaling  #3195 | rabbit | Synthetic peptide corresponding to the sequence surrounding Pro780 of human E-cadherin protein. | Monoclonal IgG | 20 |
| Pan  Neurofascin | Dr.Diane Sherman'lab* #NFC2 | rabbit | Pan Neurofasci NFC2 recognize both Nfasc155 and Nfasc186. | Polyclonal IgG | 21 |
| Caspr | NeuroMab # 75-001 | mouse | Fusion protein 1308-1381 (cytoplasmic domain) of rat Caspr | Monoclonal IgG | 22 |
| MEK1 | Cell Signaling  #9124 | rabbit | Synthetic peptide corresponding to the amino-terminus of human MEK1 | Polyclonal | 23 |
| Phospho-MEK1 (Ser144) | Cell Signaling  #9128 | rabbit | Synthetic phosphopeptide corresponding to residues surrounding Ser298 of human MEK1 | Polyclonal | 23 |
| Phospho- PAK2 (Ser20) | Cell Signaling  #2607 | rabbit | Synthetic phosphopeptide corresponding to residues surrounding Ser20 of human PAK2 | Polyclonal | 9, 24 |

**Reference list for S4 Table. Primary antibodies**

1. Veeravalli KK, Chetty C, Ponnala S, Gondi CS, Lakka SS, Fassett D, et al. MMP-9, uPAR and cathepsin B silencing downregulate integrins in human glioma xenograft cells in vitro and in vivo in nude mice. PloS one. 2010;5(7):e11583. Epub 2010/07/27. doi: 10.1371/journal.pone.0011583. PubMed PMID: 20657647; PubMed Central PMCID: PMC2904700.

2. Gurbuz I, Ferralli J, Roloff T, Chiquet-Ehrismann R, Asparuhova MB. SAP domain-dependent Mkl1 signaling stimulates proliferation and cell migration by induction of a distinct gene set indicative of poor prognosis in breast cancer patients. Mol cancer. 2014;13:22. Epub 2014/02/06. doi: 10.1186/1476-4598-13-22. PubMed PMID: 24495796; PubMed Central PMCID: PMC3933235.

3. Kriz V, Pospichalova V, Masek J, Kilander MB, Slavik J, Tanneberger K, et al. beta-arrestin promotes Wnt-induced low density lipoprotein receptor-related protein 6 (Lrp6) phosphorylation via increased membrane recruitment of Amer1 protein. J Biol Chem. 2014;289(2):1128-41. Epub 2013/11/23. doi: 10.1074/jbc.M113.498444. PubMed PMID: 24265322; PubMed Central PMCID: PMC3887180.

4. Souza BR, Romano-Silva MA, Tropepe V. Dopamine D2 receptor activity modulates Akt signaling and alters GABAergic neuron development and motor behavior in zebrafish larvae. J Neurosci. 2011;31(14):5512-25. Epub 2011/04/08. doi: 10.1523/JNEUROSCI.5548-10.2011. PubMed PMID: 21471388.

5. Miyamoto T, Morita K, Takemoto D, Takeuchi K, Kitano Y, Miyakawa T, et al. Tight junctions in Schwann cells of peripheral myelinated axons: a lesson from claudin-19-deficient mice. J Cell Biol. 2005;169(3):527-38. Epub 2005/05/11. doi: 10.1083/jcb.200501154. PubMed PMID: 15883201; PubMed Central PMCID: PMC2171943.

6. Chen FQ, Zheng HW, Hill K, Sha SH. Traumatic noise activates Rho-family GTPases through transient cellular energy depletion. J Neurosci. 2012;32(36):12421-30. Epub 2012/09/08. doi: 10.1523/JNEUROSCI.6381-11.2012. PubMed PMID: 22956833; PubMed Central PMCID: PMC3445016.

7. Lee SH, Jung YS, Chung JY, Oh AY, Lee SJ, Choi DH, et al. Novel tumor suppressive function of Smad4 in serum starvation-induced cell death through PAK1-PUMA pathway. Cell Death Dis. 2011;2:e235. Epub 2011/12/02. doi: 10.1038/cddis.2011.116. PubMed PMID: 22130069; PubMed Central PMCID: PMC3252743.

8. Redelman-Sidi G, Iyer G, Solit DB, Glickman MS. Oncogenic activation of Pak1-dependent pathway of macropinocytosis determines BCG entry into bladder cancer cells. Cancer Res. 2013;73(3):1156-67. Epub 2013/02/05. doi: 10.1158/0008-5472.CAN-12-1882. PubMed PMID: 23378476; PubMed Central PMCID: PMC3756537.

9. Elsherif L, Ozler M, Zayed MA, Shen JH, Chernoff J, Faber JE, et al. Potential compensation among group I PAK members in hindlimb ischemia and wound healing. PloS one. 2014;9(11):e112239. Epub 2014/11/08. doi: 10.1371/journal.pone.0112239. PubMed PMID: 25379771; PubMed Central PMCID: PMC4224450.

10. Licciulli S, Maksimoska J, Zhou C, Troutman S, Kota S, Liu Q, et al. FRAX597, a small molecule inhibitor of the p21-activated kinases, inhibits tumorigenesis of neurofibromatosis type 2 (NF2)-associated Schwannomas. J Biol Chem. 2013;288(40):29105-14. Epub 2013/08/21. doi: 10.1074/jbc.M113.510933. PubMed PMID: 23960073; PubMed Central PMCID: PMC3790009.

11. Oglesby IK, Bray IM, Chotirmall SH, Stallings RL, O'Neill SJ, McElvaney NG, et al. miR-126 is downregulated in cystic fibrosis airway epithelial cells and regulates TOM1 expression. J Immunol. 2010;184(4):1702-9. Epub 2010/01/20. doi: 10.4049/jimmunol.0902669. PubMed PMID: 20083669.

12. Biswas K, Yoshioka K, Asanuma K, Okamoto Y, Takuwa N, Sasaki T, et al. Essential role of class II phosphatidylinositol-3-kinase-C2alpha in sphingosine 1-phosphate receptor-1-mediated signaling and migration in endothelial cells. J Biol Chem. 2013;288(4):2325-39. Epub 2012/11/30. doi: 10.1074/jbc.M112.409656. PubMed PMID: 23192342; PubMed Central PMCID: PMC3554904.

13. Neumann M, Klar S, Wilisch-Neumann A, Hollenbach E, Kavuri S, Leverkus M, et al. Glycogen synthase kinase-3beta is a crucial mediator of signal-induced RelB degradation. Oncogene. 2011;30(21):2485-92. Epub 2011/01/11. doi: 10.1038/onc.2010.580. PubMed PMID: 21217772.

14. Musteanu M, Blaas L, Mair M, Schlederer M, Bilban M, Tauber S, et al. Stat3 is a negative regulator of intestinal tumor progression in Apc(Min) mice. Gastroenterology. 2010;138(3):1003-11 e1-5. Epub 2009/12/08. doi: 10.1053/j.gastro.2009.11.049. PubMed PMID: 19962983.

15. Cattin AL, Le Beyec J, Barreau F, Saint-Just S, Houllier A, Gonzalez FJ, et al. Hepatocyte nuclear factor 4alpha, a key factor for homeostasis, cell architecture, and barrier function of the adult intestinal epithelium. Mol Cell Biol. 2009;29(23):6294-308. Epub 2009/10/07. doi: 10.1128/MCB.00939-09. PubMed PMID: 19805521; PubMed Central PMCID: PMC2786690.

16. Hodapp JA, Carter GT, Lipe HP, Michelson SJ, Kraft GH, Bird TD. Double trouble in hereditary neuropathy: concomitant mutations in the PMP-22 gene and another gene produce novel phenotypes. Arch Neurol. 2006;63(1):112-7. Epub 2006/01/13. doi: 10.1001/archneur.63.1.112. PubMed PMID: 16401743.

17. Jin F, Dong B, Georgiou J, Jiang Q, Zhang J, Bharioke A, et al. N-WASp is required for Schwann cell cytoskeletal dynamics, normal myelin gene expression and peripheral nerve myelination. Development. 2011;138(7):1329-37. Epub 2011/03/10. doi: 10.1242/dev.058677. PubMed PMID: 21385763; PubMed Central PMCID: PMC3188810.

18. Zhang Y, Zhao Y, Jiang G, Zhang X, Zhao H, Wu J, et al. Impact of p120-catenin isoforms 1A and 3A on epithelial mesenchymal transition of lung cancer cells expressing E-cadherin in different subcellular locations. PloS one. 2014;9(2):e88064. Epub 2014/02/08. doi: 10.1371/journal.pone.0088064. PubMed PMID: 24505377; PubMed Central PMCID: PMC3913724.

19. Karjalainen M, Kakkonen E, Upla P, Paloranta H, Kankaanpaa P, Liberali P, et al. A Raft-derived, Pak1-regulated entry participates in alpha2beta1 integrin-dependent sorting to caveosomes. Mol Biol Cell. 2008;19(7):2857-69. Epub 2008/05/02. doi: 10.1091/mbc.E07-10-1094. PubMed PMID: 18448666; PubMed Central PMCID: PMC2441652.

20. Chow G, Tauler J, Mulshine JL. Cytokines and growth factors stimulate hyaluronan production: role of hyaluronan in epithelial to mesenchymal-like transition in non-small cell lung cancer. J Biomed Biotechnol. 2010;2010:485468. Epub 2010/07/31. doi: 10.1155/2010/485468. PubMed PMID: 20671927; PubMed Central PMCID: PMC2910509.

21. Tait S, Gunn-Moore F, Collinson JM, Huang J, Lubetzki C, Pedraza L, et al. An oligodendrocyte cell adhesion molecule at the site of assembly of the paranodal axo-glial junction. J Cell Biol. 2000;150(3):657-66. Epub 2000/08/10. PubMed PMID: 10931875; PubMed Central PMCID: PMC2175192.

22. Chen N, Koopmans F, Gordon A, Paliukhovich I, Klaassen RV, van der Schors RC, et al. Interaction proteomics of canonical Caspr2 (CNTNAP2) reveals the presence of two Caspr2 isoforms with overlapping interactomes. Biochim Biophys Acta. 2015;1854(7):827-33. Epub 2015/02/25. doi: 10.1016/j.bbapap.2015.02.008. PubMed PMID: 25707359.

23. Slack-Davis JK, Eblen ST, Zecevic M, Boerner SA, Tarcsafalvi A, Diaz HB, et al. PAK1 phosphorylation of MEK1 regulates fibronectin-stimulated MAPK activation. J Cell Biol. 2003;162(2):281-91. Epub 2003/07/24. doi: 10.1083/jcb.200212141. PubMed PMID: 12876277; PubMed Central PMCID: PMC2172784.

24. Zhan Q, Ge Q, Ohira T, Van Dyke T, Badwey JA. p21-activated kinase 2 in neutrophils can be regulated by phosphorylation at multiple sites and by a variety of protein phosphatases. J Immunol. 2003;171(7):3785-93. Epub 2003/09/23. PubMed PMID: 14500679.
