# Supplementary material for: Tuning PAK Activity to Rescue Abnormal Myelin Permeability in HNPP
Source: PLoS Genet. 2016 Sep 1;12(9):e1006290. doi: 10.1371/journal.pgen.1006290 (PMC5008806; doi:10.1371/journal.pgen.1006290)
Supplement: S1 References — (DOCX) [file pgen.1006290.s010.docx]

**S1 References**

1. Guo J, Wang L, Zhang Y, Wu J, Arpag S, Hu B, et al. Abnormal junctions and permeability of myelin in PMP22-deficient nerves. Ann Neurol. 2014;75(2):255-65. Epub 2013/12/18. doi: 10.1002/ana.24086. PubMed PMID: 24339129; PubMed Central PMCID: PMC4206215.

2. Cardenas AM, Allen DD, Arriagada C, Olivares A, Bennett LB, Caviedes R, et al. Establishment and characterization of immortalized neuronal cell lines derived from the spinal cord of normal and trisomy 16 fetal mice, an animal model of Down syndrome. J Neurosci Res. 2002;68(1):46-58. Epub 2002/04/05. PubMed PMID: 11933048.

3. Zou J, Hu B, Arpag S, Yan Q, Hamilton A, Zeng YS, et al. Reactivation of Lysosomal Ca2+ Efflux Rescues Abnormal Lysosomal Storage in FIG4-Deficient Cells. J Neurosci. 2015;35(17):6801-12. Epub 2015/05/01. doi: 10.1523/JNEUROSCI.4442-14.2015. PubMed PMID: 25926456; PubMed Central PMCID: PMC4412898.

4. Saavedra JT, Wolterman RA, Baas F, ten Asbroek AL. Myelination competent conditionally immortalized mouse Schwann cells. J Neurosci Methods. 2008;174(1):25-30. Epub 2008/07/29. doi: 10.1016/j.jneumeth.2008.06.029. PubMed PMID: 18657574.

5. Dyck PJ,T.P., Griffin JW, Low PA, and Poduslo JF. Peripheral Neuropathy. 1993; W.B. Saunders Co.

6. Bai Y, Zhang X, Katona I, Saporta MA, Shy ME, O'Malley HA, et al. Conduction block in PMP22 deficiency. J Neurosci. 2010;30(2):600-8. Epub 2010/01/15. doi: 10.1523/JNEUROSCI.4264-09.2010. PubMed PMID: 20071523; PubMed Central PMCID: PMC3676309.

7. Li J. Molecular regulators of nerve conduction - Lessons from inherited neuropathies and rodent genetic models. Exp Neurol. 2015;267:209-18. Epub 2015/03/21. doi: 10.1016/j.expneurol.2015.03.009. PubMed PMID: 25792482; PubMed Central PMCID: PMC4417062.
